# m^6^Am methyltransferase PCIF1 is essential for aggressiveness of gastric cancer cells by inhibiting *TM9SF1* mRNA translation

**DOI:** 10.1038/s41421-022-00395-1

**Published:** 2022-05-21

**Authors:** Wei Zhuo, Meng Sun, Kun Wang, Lu Zhang, Kai Li, Danyang Yi, Mengjie Li, Qiang Sun, Xixi Ma, Wei Liu, Lisong Teng, Chengqi Yi, Tianhua Zhou

**Affiliations:** 1 Department of Cell Biology and Department of Gastroenterology of Sir Run Run Shaw Hospital, Zhejiang University School of Medicine, Hangzhou, China; 2 State Key Laboratory of Protein and Plant Gene Research, School of Life Sciences, Peking University, Beijing, China; 3 Department of Chemical Biology and Synthetic and Functional Biomolecules Center, College of Chemistry and Molecular Engineering, Peking University, Beijing, China; 4 Cancer Center, Zhejiang University, Hangzhou, China; 5 Department of Surgical Oncology, The First Affiliated Hospital, Zhejiang University School of Medicine, Hangzhou, China; 6 Department of Molecular Genetics, University of Toronto, Toronto, ON Canada

**Keywords:** Gastric cancer, Cell invasion

## Abstract

PCIF1 (phosphorylated CTD interacting factor 1) is the first reported RNA N6,2′-O-dimethyladenosine (m^6^Am) methyltransferase. However, the pathological significance of PCIF1 and m^6^Am modification remains unknown. Here we find that both PCIF1 expression and m^6^Am modification are significantly elevated in gastric cancer tissues. Increased PCIF1 is associated with gastric cancer progression, and predicts poor prognosis. Silence of PCIF1 inhibits the proliferation and invasion of gastric cancer cells, and suppresses tumor growth and metastasis in mouse model. m^6^Am-seq analysis reveals *TM9SF1* (transmembrane 9 superfamily member 1) as a target of PCIF1. PCIF1 modifies *TM9SF1* mRNA with m^6^Am leading to decreased *TM9SF1* translation. TM9SF1 reverses the effects of PCIF1 on gastric cancer cell aggressiveness. Collectively, our work uncovers an oncogenic function of PCIF1, providing insights into the critical role of m^6^Am modification in cancer progression.

## Introduction

Emerging studies have documented that mRNA modifications play an important roles in gene expression and various biological processes^1^. Two of the most prevalent and reversible modifications within mRNAs are N6-methyladenosine (m^6^A) and N6,2′-O-dimethyladenosine (m^6^Am). The m^6^A modification mainly occurs in the internal region of mRNAs, with a marked enrichment in their 3′-UTR. The physiological significance of m^6^A modification has been well studied in past years^2,3^. m^6^Am was originally discovered at the 5′-end of mRNAs in animal cells and viruses^4^. Since fat mass-associated and obesity-associated protein (FTO) was characterized as an m^6^Am demethylase, m^6^Am has been suggested as a reversible and dynamic modification^5,6^. Nevertheless, FTO demethylates both m^6^A and m^6^Am, making it difficult to functionally separate these two modifications. Recently, we and others have independently discovered that PCIF1 (phosphorylated CTD interacting factor 1) is a cap-specific N6-methyltransferase of m^6^Am, and provides evidences that m^6^Am modification in mRNAs plays critical roles in gene regulation^7–10^. However, the role of m^6^Am modification in human cancers remains unknown.

PCIF1 was originally identified and named due to its ability to directly bind to the phosphorylated C-terminal domain (CTD) of RNA polymerase II via its WW domain^11^. PCIF1 negatively regulates gene expression by modulating the phosphorylation status of RNA polymerase II^12^. In addition, PCIF1 has been reported to inhibit the expression of pancreatic-duodenal homeobox 1 (PDX-1) that is critical for normal pancreas development through its conserved TRAF and POZ domains^13,14^. Further study shows that PCIF1 limits PDX-1 protein accumulation and modulates the function and survival of pancreatic β cells in mice^15^. However, the pathological significance of PCIF1 in human cancers has not been investigated.

Gastric cancer is the fifth most common malignancy with the fourth highest mortality rate worldwide and has a high prevalence in East Asia^16,17^. Gastric cancer patients are often in advanced stages at the time of consultation and the 5-year survival rate of those patients are lower than 25%^18^. Therefore, developing effective strategy to treat gastric cancer is an urgent medical need, which requires further deep exploration of the molecular mechanisms of gastric carcinogenesis.

In this study, we identified that PCIF1 and m^6^Am modification levels in mRNAs are significantly upregulated in gastric cancer tissues. PCIF1 predicts a poor prognosis and plays an oncogenic role in gastric cancer development. Moreover, we found that *TM9SF1* (transmembrane 9 superfamily member 1) is a functional mRNA target of PCIF1 and acts as a tumor suppressor gene in gastric cancer. The PCIF1-mediated m^6^Am modification of *TM9SF1* mRNA decreases the translational efficiency of *TM9SF1*. Taken together, our data suggest the crucial roles of PCIF1 and m^6^Am modification in gastric cancer progression.

## Results

### PCIF1 is upregulated in gastric cancer tissues and associated with poor survival

**Fig. 1:**
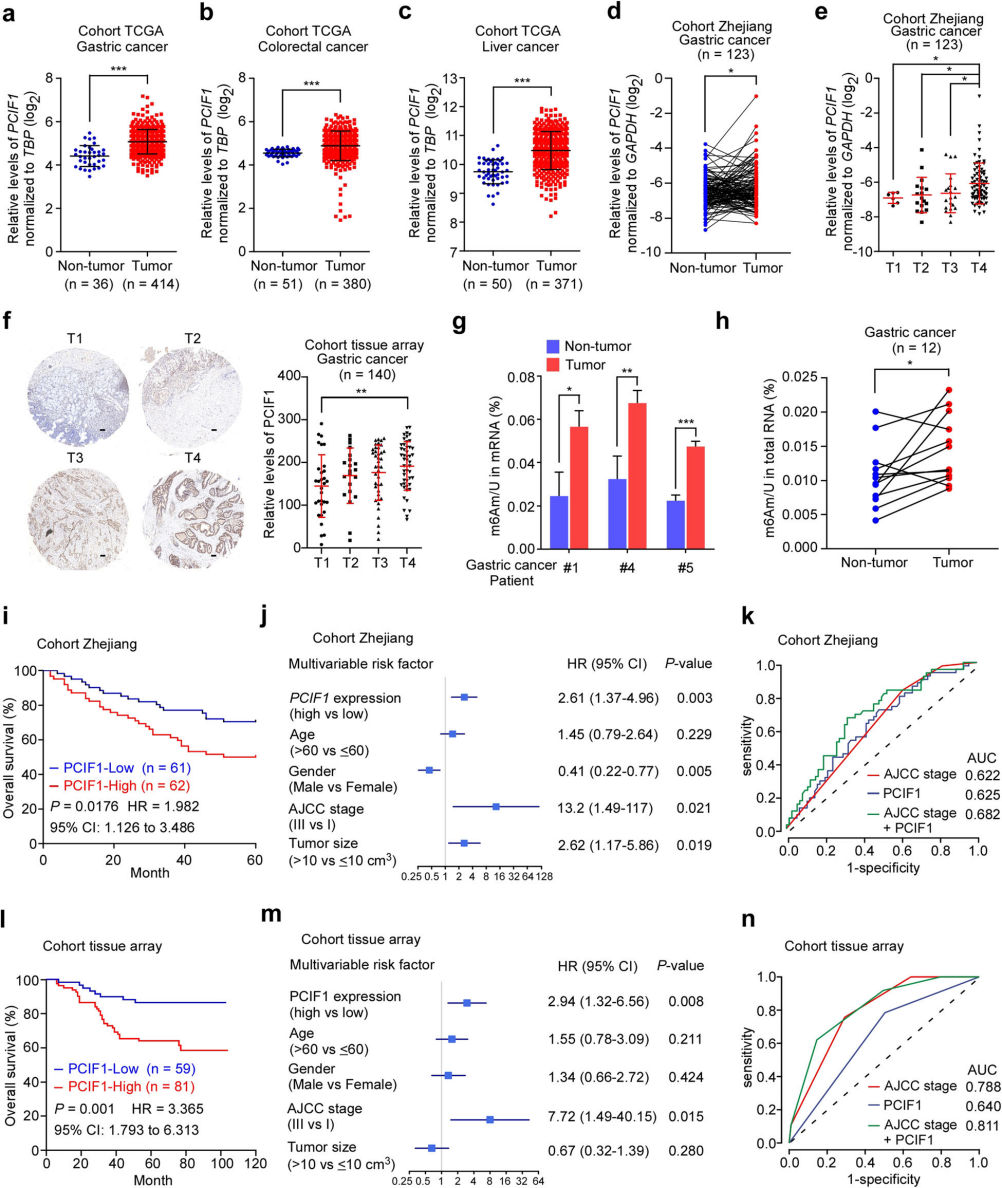
PCIF1 is significantly upregulated in gastric cancer tissues and associated with poor survival. **a**–**c** The expression of *PCIF1* mRNA in various human cancers was analyzed by using TCGA database. *PCIF1* expression in gastric (**a**), colorectal (**b**), and liver cancers (**c**) compared to their corresponding non-tumor tissues are shown. Data are presented as log_2_ value of *PCIF1* relative to *TBP* (TATA binding protein). **d**, **e** The levels of *PCIF1* mRNA in gastric cancer tissues and their adjacent non-tumor tissues from cohort Zhejiang was detected by quantitative RT-PCR. The expression pattern of *PCIF1* mRNA in gastric cancer tissues from patients with different tumor stages is shown. Data are expressed as log_2_ value of *PCIF1* relative to *GAPDH*. **f** Immunohistochemistry analysis of gastric cancer tissue array probed with anti-PCIF1 antibody. Representative images from gastric cancer patients with different tumor stages (cohort tissue array) are presented. Immunohistochemistry scores were also analyzed. Scale bars, 200 μm. **g** LC-MS/MS quantification of the m^6^Am/U ratios of mRNAs from gastric cancer tissues and their adjacent non-tumor tissues. **h** LC-MS/MS quantification of the m^6^Am/U ratios of total RNAs from gastric cancer tissues and their corresponding non-tumor tissues. **i** Kaplan–Meier survival curves of *PCIF1* mRNA expression in gastric cancer tissues from cohort Zhejiang with best cutoff. **j** Multivariable risk factor analyses of cohort Zhejiang. All the bars correspond to 95% confidence intervals (CI). HR hazard rate, AJCC American Joint Committee on Cancer. **k** Comparison of predictive values in gastric cancer patients based on *PCIF1* mRNA levels, AJCC stages, and their combination by ROC analysis in cohort Zhejiang. **l** Kaplan–Meier survival curves of PCIF1 protein expression in cohort tissue array with best cutoff. **m** Multivariable risk factor analyses of cohort tissue array. All the bars correspond to 95% CI. HR hazard rate. **n** Comparison of predictive values in gastric cancer patients based on PCIF1 levels, AJCC stages, and their combination by ROC analysis in cohort tissue array. Data are shown as means ± SD. *P < 0.05; **P < 0.01, ***P < 0.001; Mann–Whitney test (**a**–**c**, **e**, **f**), paired *t*-test (**d**, **h**), Student’s *t-*test (**g**), the log-rank test (**k**, **n**).

To explore the clinical relevance of the m^6^Am methyltransferase PCIF1 in cancers, we queried the Cancer Genome Atlas (TCGA) datasets. Our data revealed that the expression level of *PCIF1* mRNA was significantly elevated in tumor tissues compared with their corresponding non-tumor tissues in various human cancers (Supplementary Fig. S1), including gastric, colorectal, and liver cancers (Fig. 1a–c). Quantitative RT-PCR (qRT-PCR) confirmed that *PCIF1* mRNA was significantly increased in tumor tissues from 123 gastric cancer patients (cohort Zhejiang) (Fig. 1d). *PCIF1* mRNA levels were higher in tumor tissues from patients with advanced gastric cancer than that of patients in early stages (Fig. 1e). Furthermore, we examined PCIF1 protein expression in paraffin-embedded tumor tissues from 140 gastric cancer patients (cohort tissue array) (Fig. 1f). Immunohistochemical staining showed that PCIF1 protein was also significantly raised in tumor tissues form patients with T4 stage compared to that of T1 stage. Thus, these data indicate that PCIF1 is significantly upregulated in gastric cancer tissues and associated with gastric cancer development.

Given that PCIF1 has been reported as an mRNA m^6^Am methyltransferase^7–10^, we tried to test the m^6^Am modification levels of mRNAs in gastric cancer tissues. Quantitative mass spectrometry results showed that the m^6^Am levels in mRNAs from tumor tissues were significantly upregulated compared with that from their adjacent non-tumor tissues (Fig. 1g). Additionally, we isolated total RNAs and observed the similar increase of their m^6^Am levels in gastric cancer tissues compared with paired non-tumor tissues (Fig. 1h). These findings suggest that the RNA m^6^Am modification is significantly elevated in gastric cancer tissues.

The significant upregulation of PCIF1 levels in gastric cancer tissues prompted us to determine the correlation between PCIF1 expression and clinical outcomes. Kaplan–Meier plotter analysis of cohort Zhejiang revealed that patients with increased *PCIF1* mRNA expression had poor overall survival (Fig. 1i). Multivariate COX analysis showed that high *PCIF1* expression was an independent negative prognostic factor for predicting clinical outcome of gastric cancer patients (Fig. 1j). Kaplan–Meier and multivariate COX analyses of cohort ACRG (Asian Cancer Research Group, GSE62254) confirmed that *PCIF1* mRNA expression was also an independent poor prognostic factor for gastric cancer patients (Supplementary Fig. S2). To evaluate the prognostic value of *PCIF1* mRNA expression, we performed receiver operating characteristic (ROC) curve analysis, and found that area under curve (AUC) of the combination of *PCIF1*-based prediction and American Joint Committee on Cancer (AJCC)-based prediction was higher than that of AJCC alone (Fig. 1k). These results were further verified by analyzing the clinical value of PCIF1 protein levels in tissue array cohort (Fig. 1l–n). Taken together, our data suggest that elevated expression of PCIF1 in gastric cancer tissues is an independent predictor for poor prognosis.

### Knockdown of PCIF1 inhibits the aggressiveness of gastric cancer cells

**Fig. 2:**
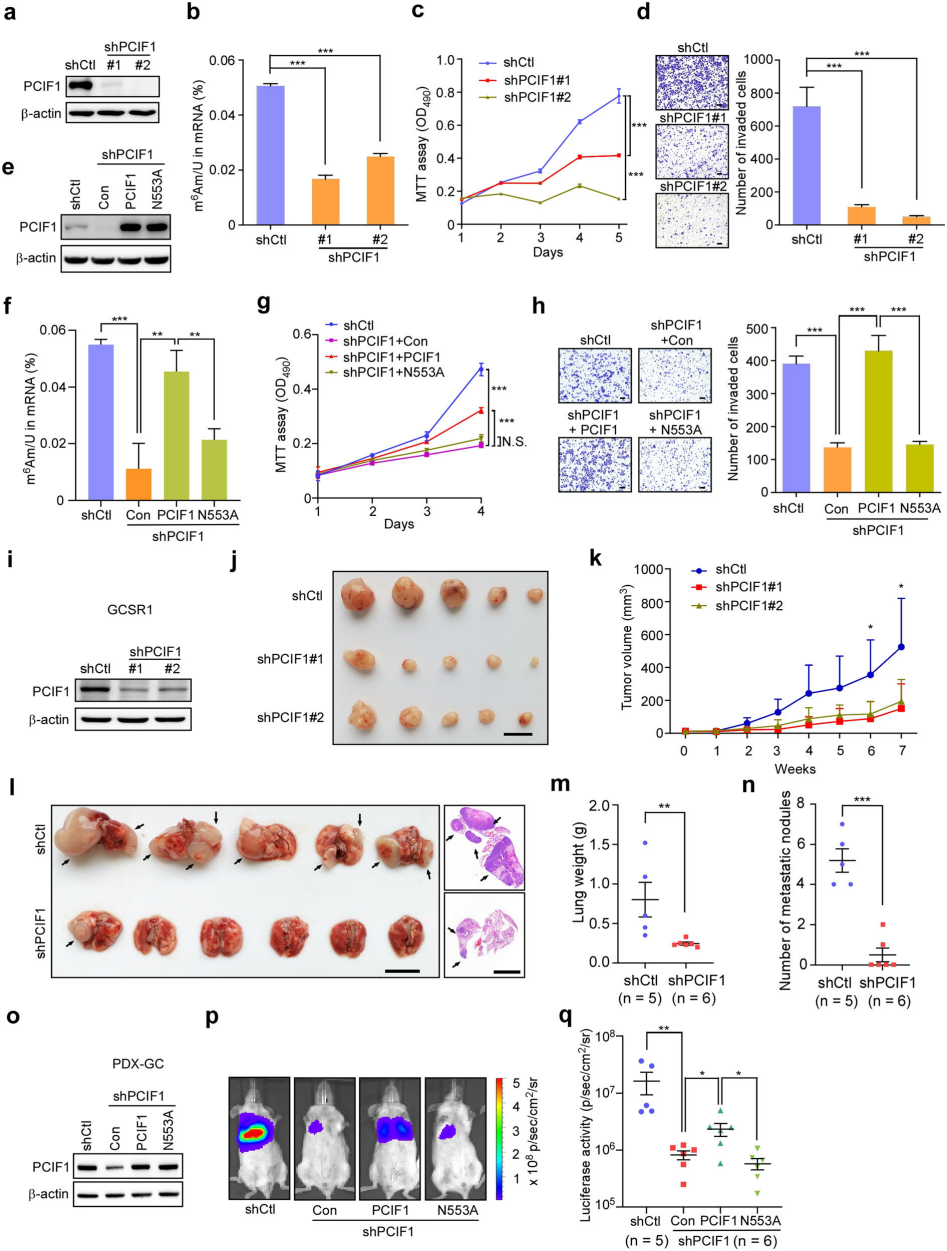
Knockdown of PCIF1 inhibits the aggressiveness of gastric cancer cells. **a** Confirmation of PCIF1 knockdown in AGS cells with two independent lentivirus-based PCIF1 shRNAs by western blot analysis. **b** LC-MS/MS quantification of the m^6^Am/U ratios of mRNAs in control and PCIF1-depleted AGS cells. **c** MTT assays of AGS cells with control or PCIF1 shRNAs. **d** Migration analysis of AGS cells with control or PCIF1 shRNAs. Representative images of invaded cells are shown. The invaded cells were counted. Scale bars, 100 μm. **e** Western blot analysis of PCIF1 expression in control AGS cells, PCIF1-depleted cells, and PCIF1-depleted cells transfected with wild-type or mutant PCIF1 (N553A) plasmid. **f** LC-MS/MS quantification of the m^6^Am/U ratios of mRNAs in AGS cells with the indicated treatments. **g** MTT assays of AGS cells with the indicated shRNA and plasmids. **h** Migration analysis of AGS cells with the indicated shRNAs and plasmids. Representative images of invaded cells are presented. The invaded cells were quantified. Scale bars, 100 μm. **i** Confirmation of PCIF1 depletion in GCSR1 cells with lentivirus-based control or PCIF1 shRNAs by western blotting. **j**, **k** Control and PCIF1-depleted GCSR1 cells were subcutaneously injected into BALB/C nude mice. The tumor volume was measured every week, and the growth curves are shown. The images of tumors in each group are presented (*n* = 5). Scale bar, 1 cm. **l**–**n** Control and PCIF1-knockdown GCSR1 cells were intravenously injected into SCID mice and lung metastases (black arrows) were measured. The lung was weighted at the end of experiment. Gross lung and representative H&E images are presented (**l**). Statistics analyses of lung weight (**m**) and metastatic nodule number (**n**) are shown. Scale bars, 1 cm. **o**–**q** PDX-GC cells stably expressed a luciferase reporter gene were infected by lentivirus-based shRNA targeting PCIF1, and then overexpressed with wild-type PCIF1 or its catalytically inactive N553A mutant. Western blotting with the indicated antibodies is shown (**o**). These PDX-GC cells were intravenously injected into SCID mice. Representative bioluminescence images are also presented (**p**). Quantification analyses of bioluminescent images of lung metastases are shown (**q**). Data are expressed as means ± SD. *P < 0.05, **P < 0.01, ***P < 0.001, Student’s *t-*test (**b**–**d**, **f**–**h**, **k**), Mann–Whitney test (**m**, **n**, **q**).

To explore the biological roles of PCIF1 in gastric cancer progression, we depleted PCIF1 expression in AGS cells with two different lentivirus-based shRNAs (Fig. 2a). The m^6^Am levels of global mRNAs were significantly decreased in PCIF1-depleted cells compared with control cells (Fig. 2b). At the same time, PCIF1 depletion dramatically reduced the proliferation and invasion of AGS cells (Fig. 2c, d). Similar results were also observed in BGC-823 cells (Supplementary Fig. S3). To determine whether PCIF1 plays oncogenic function via its m^6^Am methyltransferase activity, we generated a catalytically inactive PCIF1 mutant by mutating asparagine 553 to alanine (N553A), a key residue for the m^6^Am methyltransferase activity of PCIF1^7,10^. Exogenous expression of wild-type PCIF1, but not its N553A mutant, was able to rescue the m^6^Am levels of global mRNAs in PCIF1-depleted cells (Fig. 2e, f). Importantly, ectopic expression of wild-type PCIF1, rather than N553A mutant, reversed the inhibitory effects of cell proliferation and invasion induced by PCIF1 knockdown (Fig. 2g, h). Collectively, these results suggest that PCIF1 is essential for gastric cancer cell proliferation and invasion.

To further evaluate the role of PCIF1 in gastric carcinogenesis in vivo, a gastric cancer cell line GCSR1 directly derived from patient-derived xenograft (PDX) was employed^19^. PCIF1 was successfully depleted by two lentivirus-based shRNAs in GCSR1 cells (Fig. 2i). Subcutaneous implantation analysis of cancer cells in nude mice showed that knockdown of PCIF1 in GCSR1 cells obviously suppressed tumor growth compared with control cells (Fig. 2j, k). Then, we intravenously implanted control and PCIF1-depleted GCSR1 cells to conduct lung metastasis assay as reflected by gross lung image, H&E staining, lung weight, and metastatic nodules (Fig. 2l–n). Our results showed that mice received control GCSR1 cells developed obvious metastatic nodules in lung. Silencing of PCIF1 robustly inhibited the lung metastasis of GCSR1 cells. Moreover, we established a new gastric cancer PDX cell line (PDX-GC), which stably expressed a luciferase reporter gene. PDX-GC cells were infected by lentivirus-based shRNA targeting PCIF1, and further overexpressed with wild-type PCIF1 or its catalytically inactive N553A mutant (Fig. 2o). These PDX-GC cells were then subjected to pulmonary metastasis assays (Fig. 2p, q). Our results showed that exogenous expression of wild-type PCIF1, rather than N553A mutant, reversed the metastasis inhibition induced by PCIF1 knockdown. Taken together, these data indicate that PCIF1 functions as an oncogene in gastric cancer.

### Identification of *TM9SF1* as a potential target mRNA of PCIF1

**Fig. 3:**
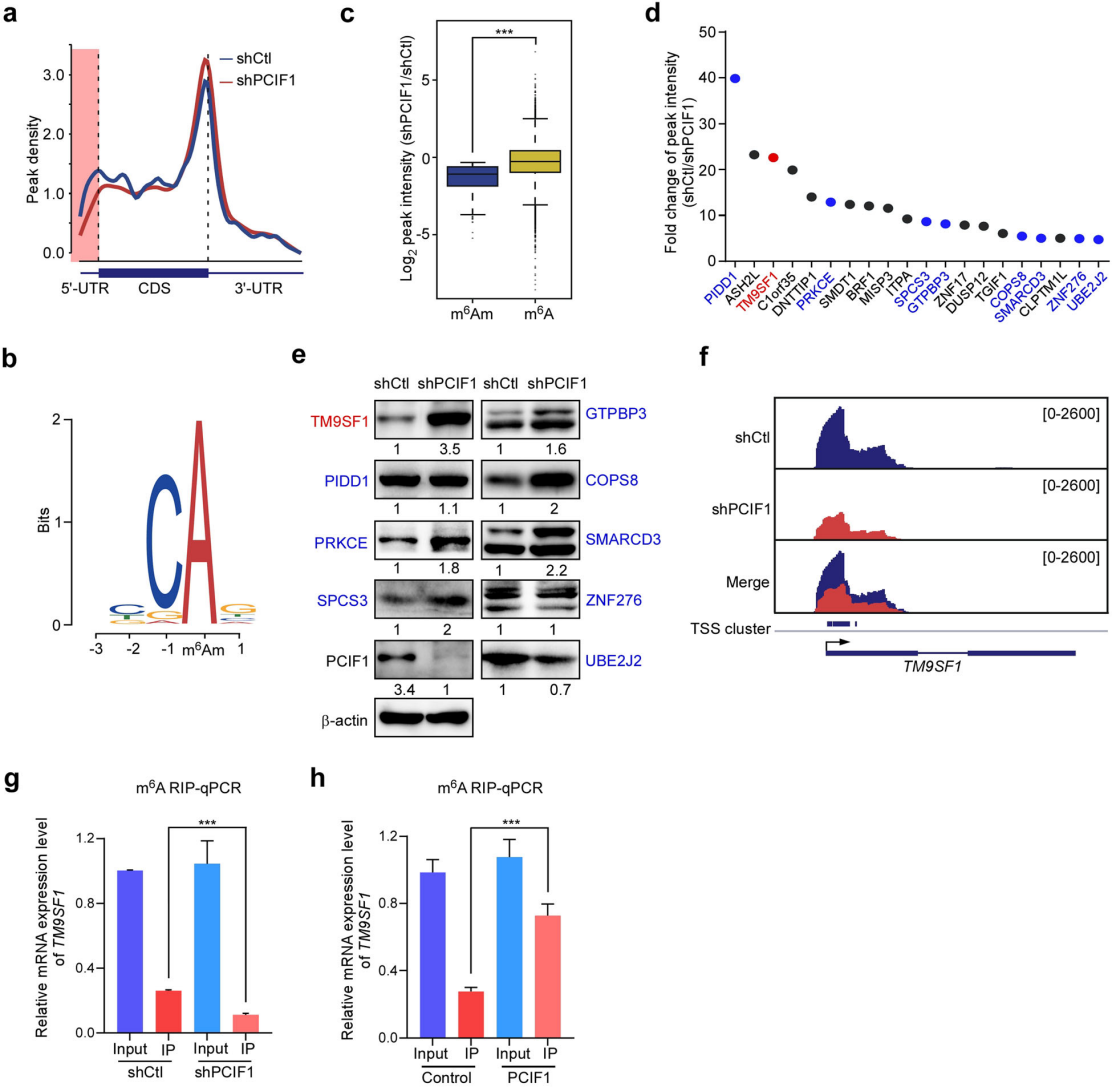
Identification of *TM9SF1* as a target mRNA of PCIF1. **a**–**c** Transcriptome-wide m^6^A-seq analyses of control or PCIF1-depleted AGS cells. Distribution of m^6^A/m^6^Am peaks across mRNA segments in AGS cells (**a**). Each segment was normalized according to its average length by RefSeq annotation. Motif analysis of m^6^Am peaks of mRNAs modified by PCIF1 in AGS cells (**b**). BCA, A = m^6^Am; B = C, G, or U. *E*-value = 4.5e−54. Boxplot analysis of change of m^6^A/m^6^Am peaks intensity in AGS cells upon PCIF1 knockdown is shown (**c**). **d** m^6^A-Seq analysis of top 20 down-regulated genes in m^6^Am abundance in AGS cells. The data are presented by the fold change of peak intensity (shControl/shPCIF1). ASH2L ASH2 (absent, small, or homeotic)-like, BRF1 BRF1 RNA polymerase III transcription initiation factor subunit, C1orf35 chromosome 1 open reading frame 35, CLPTM1L CLPTM1 (cleft lip and palate transmembrane protein 1)-like, COPS8 COP9 signalosome subunit 8, DNTTIP1 deoxynucleotidyltransferase terminal interacting protein 1, DUSP12 dual specificity phosphatase 12, GTPBP3 GTP binding protein 3, mitochondrial; ITPA inosine triphosphatase, MISP3 MISP family member 3, PIDD1 p53-induced death domain protein 1, PRKCE protein kinase C epsilon, SMARCD3 SWI/SNF related, matrix associated, actin dependent regulator of chromatin, subfamily d, member 3, SMDT1 single-pass membrane protein with aspartate rich tail 1, SPCS3 signal peptidase complex subunit 3, TGIF1 TGFB induced factor homeobox 1, TM9SF1 transmembrane 9 superfamily member 1, UBE2J2 ubiquitin conjugating enzyme E2 J2, ZNF17 zinc finger protein 17, ZNF276 zinc finger protein 276. **e** Western analysis of the potential target genes of PCIF1 predicted by m^6^A-Seq and Kaplan–Meier survival curves (Supplementary Fig. S4) in control and PCIF1-depleted AGS cells. And intensities of PCIF1 potential target genes in PCIF1-depleted cells compared to those in control cells are shown. **f** The analysis of m^6^A/m^6^Am peaks on *TM9SF1* gene in control and PCIF1-depleted AGS cells. **g**, **h** m^6^A-RIP analysis of *TM9SF1* mRNA in AGS cells transfected with the indicated shRNA and plasmids. The enrichment of *TM9SF1* mRNA in immunoprecipitation (IP) or input fraction was determined by qRT-PCR. Data are shown as means ± SD. N.S. means not significant; ***P < 0.001, Student’s *t*-test.

To determine downstream targets of PCIF1, we carried transcriptome-wide m^6^A-seq and RNA-seq upon PCIF1 knockdown in gastric cancer cells as previously reported^20^. Given that anti-m^6^A antibody recognizes both m^6^Am and m^6^A, RNAs modified by m^6^Am or m^6^A can be enriched and detected simultaneously. We found the enrichment of m^6^Am or m^6^A modification on the transcription start site (TSS) and the stop codon regions of mRNAs in AGS cells (Fig. 3a). A specific reduction of modification peak at the TSS region was observed in PCIF1-depleted AGS cells, suggesting a possible PCIF1-dependent m^6^Am methylation^8,10^. Based on the obvious decrease of m^6^Am methylation upon PCIF1 depletion, we found 446 PCIF1-dependent m^6^Am-marked genes in AGS cells (Supplementary Table S1). A motif analysis of the genomic context of the m^6^Am peaks revealed the canonical BCA motif (A = m^6^Am; BC representing upstream genomic nucleotides; B = C, G, or U) (Fig. 3b), which was consistent with the previous report^8,20^. Moreover, the enrichment of m^6^Am peaks was significantly decreased upon PCIF1 knockdown, while the enrichment of m^6^A peaks was mildly affected (Fig. 3c), implying that PCIF1 depletion specifically reduces m^6^Am methylation.

To identify PCIF1 targets that link the m^6^Am modification of mRNAs to gastric carcinogenesis, we ranked top 20 mRNAs of the m^6^Am-modified transcripts based on the fold change of peak intensity upon PCIF1 depletion (Fig. 3d). Kaplan–Meier analysis of these PCIF1 target transcripts revealed that nine mRNAs were significantly associated with overall survival of gastric cancer patients (Supplementary Fig. S4). Among them, western blotting displayed that TM9SF1 was the most robustly increased protein in PCIF1-depleted cells (Fig. 3e). Bioinformatics analysis confirmed that *TM9SF1* mRNA contained a high confident, PCIF1-dependent m^6^Am site (Fig. 3f and Supplementary Fig. S5). To validate *TM9SF1* as a target mRNA of PCIF1, we employed RNA immunoprecipitation (RIP)-qPCR assay with anti-m^6^A antibody. Our results revealed that the relative proportion of modified *TM9SF1* mRNA was significantly reduced in PCIF1-depleted cells (Fig. 3g). Considering PCIF1 was frequently upregulated in gastric cancer tissues, we overexpressed PCIF1 and found that the relative proportion of modified *TM9SF1* mRNA was robustly increased to more than 70% in AGS cells (Fig. 3h). Collectively, these results suggest that TM9SF1 may be a downstream target of PCIF1.

### m^6^Am modification suppresses TM9SF1 expression at the translation level

**Fig. 4:**
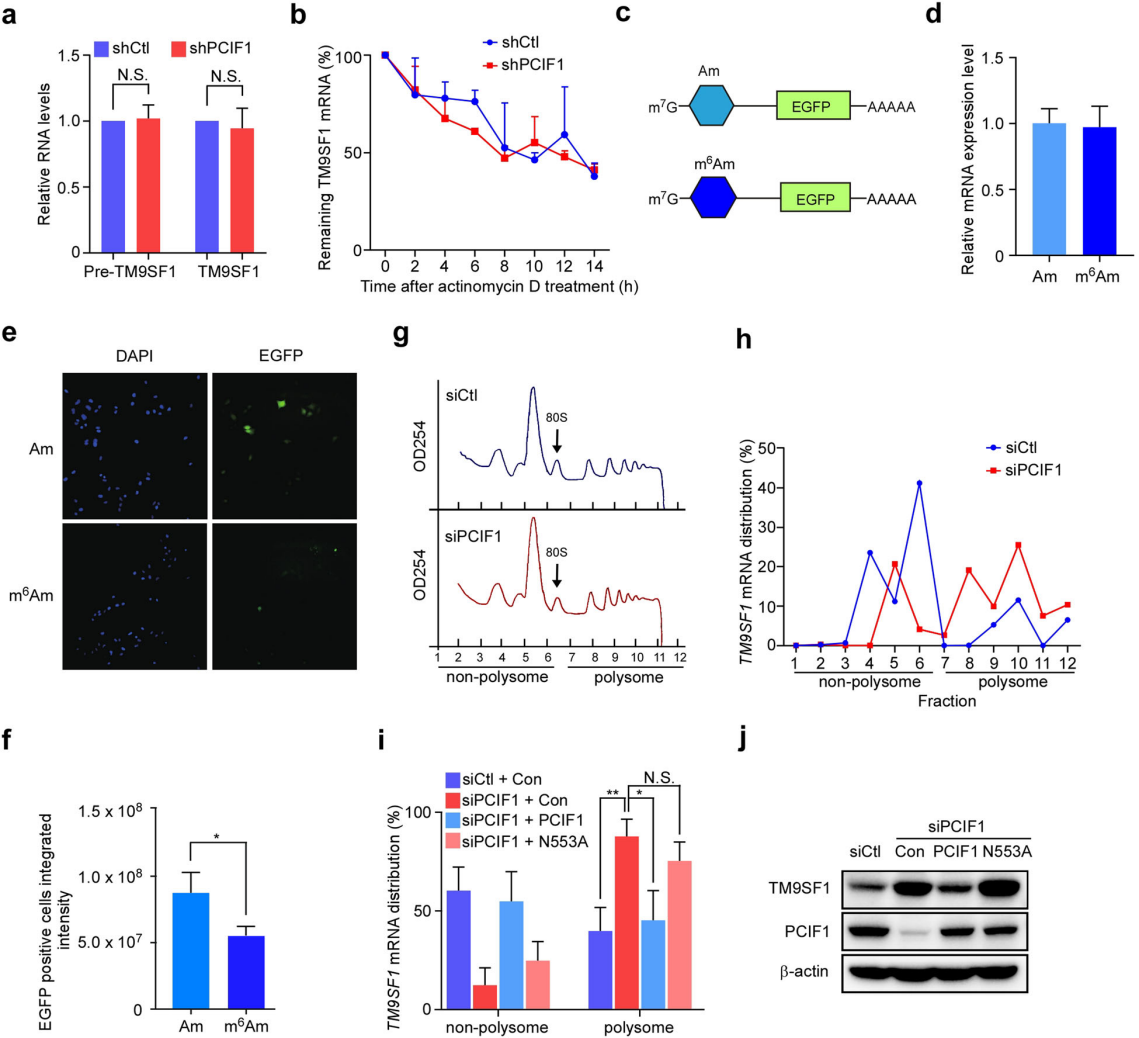
m^6^Am modification suppresses *TM9SF1* mRNA translation. **a** Quantitative RT-PCR analysis of precursor or mature *TM9SF1* mRNA expression in AGS cells with control or PCIF1 depletion. Data are presented as log_2_ value of *PCIF1* relative to *GAPDH*. **b** Analysis of remaining *TM9SF1* mRNA at the indicated times in control and PCIF1-depleted AGS cells after actinomycin D treatment. **c**–**f** The expression of m^7^G-Am or m^7^G-m^6^Am *EGFP* mRNA in Hela cells. *EGFP* mRNA that starts with either m^7^G-Am or m^7^G-m^6^Am modification was generated by in vitro transcription (**c**). Cells were transfected with m^7^G-Am or m^7^G-m^6^Am *EGFP* mRNA and subjected to qRT-PCR (**d**) and fluorescence microscopy (**e**). Representative images of EGFP in cells are shown. Fluorescence intensity of EGFP-positive Hela cells was quantified (**f**). DNA was visualized by DAPI. **g**, **h** Polysome profiling analysis of *TM9SF1* mRNA. The distribution of *TM9SF1* mRNA in each fraction of control and PCIF1-depleted BGC-823 cells was determined by qRT-PCR. **i** Analysis of *TM9SF1* mRNA distribution in the fractions of non-polysome and polysome from BGC-823 cells with the indicated siRNA and plasmids. **j** Western blot analysis of TM9SF1 in AGS cells with the indicated shRNA and plasmids. Data are shown as means ± SD. N.S. not significant; *P < 0.05, **P < 0.01, Student’s *t*-test.

To explore the effects of m^6^Am modification on gene expression, we examined the mRNA levels of m^6^Am-containing transcripts in control and PCIF1 knockdown cells (Supplementary Fig. S6). Based on the identity of the first nucleotide of these transcripts, we classified the transcripts into five groups (m^6^Am, Am, Um, Cm, and Gm) to analyze their steady-state levels. The data showed there was no significant difference of global mRNA levels upon PCIF1 knockdown (Supplementary Fig. S6), suggesting that PCIF1-mediated m^6^Am modification may not globally affect mRNA levels. Among them, the expression of *TM9SF1* precursor RNA and mature mRNA was also not significantly affected by depletion of PCIF1 (Fig. 4a). Treatment with actinomycin D to block transcription revealed that the half-life of *TM9SF1* mRNA had no significant change in cells depleted of PCIF1 (Fig. 4b). These data indicate that PCIF1 depletion does not significantly alter the transcription or mRNA stability of *TM9SF1*.

To test whether m^6^Am modification regulates gene expression at the translation level, we performed EGFP reporter assay by using mRNA with either m^7^G-Am or m^7^G- m^6^Am cap (Fig. 4c). Our results revealed that cells transfected with EGFP mRNA starting with an m^7^G-m^6^Am cap had reduced GFP signals without mRNA change compared to that with m^7^G-Am cap (Fig. 4d–f), implying that m^6^Am modification may inhibit mRNA translation. Next, we performed polysome profiling analysis, and found that PCIF1 depletion did not affect the global profiles of polysome distribution (Fig. 4g). Further experiments showed that PCIF1 knockdown induced an obvious shift of *TM9SF1* mRNA towards polysome portion (Fig. 4h), indicating a role of PCIF1 in the regulation of *TM9SF1* mRNA translation. Moreover, we reintroduced wild-type or mutant PCIF1 (N553A) into PCIF1-depleted cells for polysome profiling analysis, and found that *TM9SF1* mRNA in the polysome fraction was almost reversed by ectopic expression of wild-type PCIF1 but not mutant PCIF1 (Fig. 4i), which was consistent with western blot analysis (Fig. 4j). Together, these findings suggest an inhibitory effect of PCIF1-mediated m^6^Am modification on *TM9SF1* mRNA translation.

### TM9SF1 functions as a tumor suppressor gene in gastric cancer

**Fig. 5:**
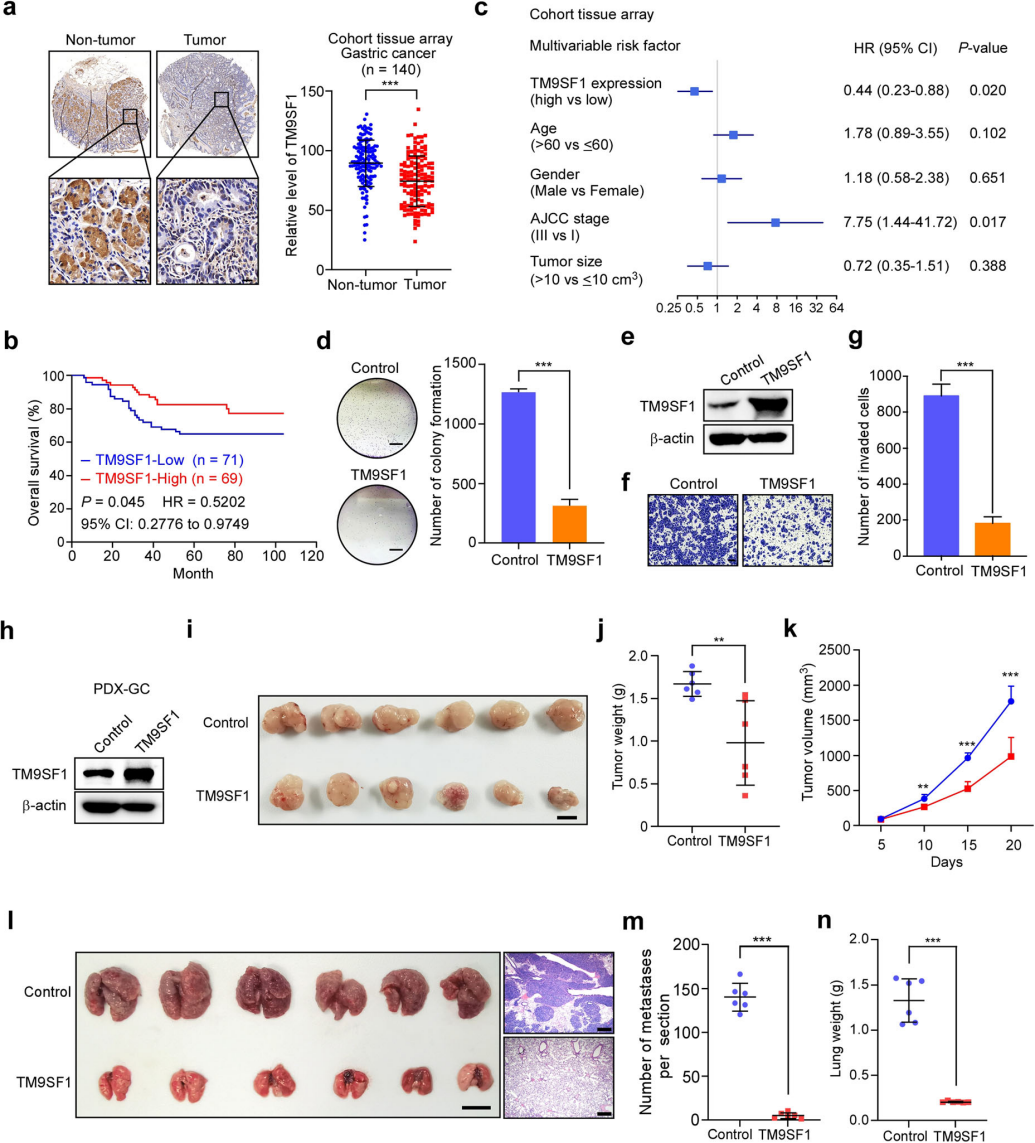
TM9SF1 functions as a tumor suppressor gene in gastric cancer. **a** Immunohistochemistry analysis of gastric cancer tissue array probed with anti-TM9SF1 antibody. Representative images from gastric cancer tissues and their pair non-tumor tissues are presented. Immunohistochemistry scores were analyzed. Bars, 20 μm. **b** Kaplan–Meier survival curves of TM9SF1 protein expression in gastric cancer tissues from cohort tissue array with best cutoff. **c** Multivariable risk factor analyses of cohort tissue array. All the bars correspond to 95% CI. HR hazard rate. **d** Colony formation assays of BGC-823 cells infected with control or TM9SF1 expression lentivirus. Representative images of cell colonies are shown. The colonies were quantified. Scale bars, 5 mm. **e** Confirmation of TM9SF1 overexpression in BGC-823 cells with control or TM9SF1 expression lentivirus by western blot analysis. **f**, **g** Migration analysis of BGC-823 cells infected with control or TM9SF1 expression lentivirus. Representative images of invaded cells are presented (**f**). The invaded cells were counted (**g**). Scale bars, 100 μm. **h**–**k** PDX-GC cells infected with control or TM9SF1 expression lentivirus were subcutaneously injected into BALB/C nude mice. The expression of TM9SF1 protein was determined by western blotting (**h**). The images of tumors in each group are shown (**i**). The average weight of tumors in each group was also analyzed (**j**). The tumor volume was measured every 5 days, and the growth curves were generated (**k**). Scale bar, 1 cm. **l**–**n** PDX-GC cells were overexpressed with TM9SF1 and intravenously injected into SCID mice. Gross lungs (Scale bars, 1 cm) and representative H&E images (Scale bars, 400 μm) are presented (**l**). Statistics analyses of pulmonary metastases (**m**) and lung weight (**n**) are shown. Data are expressed as means ± SD. **P < 0.01, ***P < 0.001, paired *t*-test (**a**), Mann–Whitney test (**j**, **m**, **n**), Student’s *t*-test (**d**, **g**, **k**).

To explore whether TM9SF1 plays a role in gastric cancer progression, we tested the expression of TM9SF1 by tissue array analysis. The results showed that TM9SF1 was significantly downregulated in gastric cancer tissues compared with their paired non-tumor tissues (Fig. 5a). Furthermore, lower expression of TM9SF1 was significantly associated with poor overall survival of gastric cancer patients (Fig. 5b). Multivariate COX analysis revealed that TM9SF1 expression was an independent favorable predictor for clinical outcome of gastric cancer patients (Fig. 5c). Together, these data imply that TM9SF1 may play a tumor suppressor role in gastric cancer.

To characterize the function of TM9SF1 in gastric cancer development, we forced to express TM9SF1 in gastric cancer cells, and found ectopic TM9SF1 significantly inhibited cell proliferation and invasion (Fig. 5d–g). Moreover, PDX-GC cells were overexpressed with TM9SF1, and then subcutaneously injected into nude mice. The result showed that exogenous expression of TM9SF1 in PDX cells significantly inhibited tumor growth (Fig. 5h–k). Besides, we intravenously implanted control and TM9SF1-overexpressed PDX-GC cells to perform lung metastasis assay. Our result indicated that exogenic expression of TM9SF1 in PDX-GC cells obviously inhibited tumor metastasis (Fig. 5l–n). Taken together, these data indicate that TM9SF1 is a tumor suppressor of gastric cancer and predicts a favorable survival of patients.

### Silencing of TM9SF1 reverses the phenotype induced by PCIF1 depletion

**Fig. 6:**
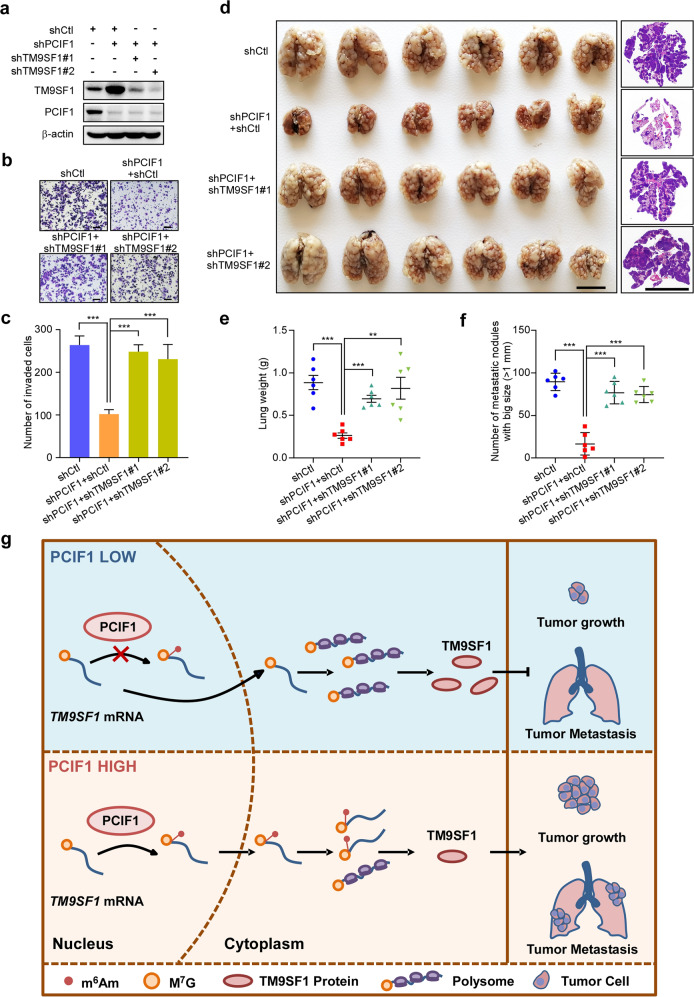
TM9SF1 depletion reverses PCIF1 knockdown-induced phenotypes. **a** Western blot analysis of TM9SF1 in BGC-823 cells infected with the indicated lentivirus-based shRNAs. **b**, **c** Migration analysis of BGC-823 cells with the indicated lentivirus-based shRNAs. Representative images of invaded cells are shown (**b**). The invaded cells were quantified (**c**). Scale bars, 100 μm. **d**–**f** BGC-823 cells with the indicated lentivirus-based shRNAs were intravenously injected into SCID mice, respectively. Representative images of gross lungs and H&E-stained lung are shown (**d**). The lung weight (**e**) and the number of metastatic nodules (>1 mm) (**f**) were quantified. Scale bars, 1 cm. **g** The working model of PCIF1-dependent m^6^Am modification of *TM9SF1* mRNA to promote gastric cancer progression. When PCIF1 level is high, it modifies *TM9SF1* mRNA with m^6^Am leading to decreased translational efficiency of *TM9SF1*. The downregulated protein level of TM9SF1 enhance gastric cancer cell malignancy. And depletion of PCIF1 promotes the translation of its target mRNA *TM9SF1*. Ectopic TM9SF1 inhibits tumor growth and metastasis of gastric cancer cells. Data are shown as means ± SD. **P < 0.01, ***P < 0.001, Student’s *t-*test (**c**), Mann–Whitney test (**e**, **f**).

Since PCIF1 is associated with gastric cancer aggressiveness and suppresses *TM9SF1* mRNA translation, we continued to determine whether TM9SF1 mediates the oncogenic role of PCIF1 in gastric cancer progression. We silenced TM9SF1 in PCIF1-depleted gastric cancer cells with two independent shRNAs, and found that knockdown of TM9SF1 significantly reversed the inhibitory effects of PCIF1 depletion on cell invasion (Fig. 6a–c) and cell proliferation (Supplementary Fig. S7). In agreement with the results above, mice received control cells developed severe lung metastasis, while mice received PCIF1-depleted cells exhibited significant reduction in the size of metastatic lesions and the number of metastatic nodules (diameter > 1 mm), supporting an important role of PCIF1 in gastric cancer metastasis (Fig. 6d–f). Additionally, knockdown of TM9SF1 in PCIF1-depleted cells significantly reversed the inhibition of metastasis induced by PCIF1 depletion (Fig. 6d–f). Taken together, these data indicate that TM9SF1 is a functional downstream target of PCIF1 during gastric cancer development.

## Discussion

m^6^Am is a prevalent RNA modification in mRNAs, which has been reported to be catalyzed by PCIF1 in mammalian cells^7–10^. However, the biological role of m^6^Am modification and PCIF1 in human cancers is largely unknown. Here, we provide evidences that m^6^Am modification and PCIF1 play a hitherto uncharacterized role in cancer progression. Our data show that m^6^Am modification and PCIF1 expression are significantly increased in gastric cancer tissues. The upregulation of PCIF1 in stomach tumor tissues is an independent predictor for poor prognosis. Depletion of PCIF1 inhibits the proliferation and invasion of gastric cancer cells, and suppresses tumor growth and metastasis. Mechanically, PCIF1 modifies *TM9SF1* mRNA with m^6^Am to inhibit its mRNA translation, which reduces the protein levels of TM9SF1 to enhance gastric cancer cell malignancy (Fig. 6g).

m^6^Am modification has been initially reported to increase mRNA stability^5,9^. Further independent studies reveal that m^6^Am modification modulates the translation of target mRNAs, but has no significant effect on mRNA stability^7,8^, which is confirmed by our data. We integrate multiple approaches, including EGFP reporter assays, polysome profiling, m^6^Am-seq and western blotting, and find that the protein levels of some highly confident m^6^Am-contaning transcripts are increased in gastric cancer cells depleted of PCIF1. Moreover, knockdown of PCIF1 obviously promotes the translation of its target mRNA *TM9SF1*. Further study is undoubtedly considered necessary to explore how m^6^Am marks regulate their target mRNA translation.

Our bioinformatics analysis reveals that PCIF1 expression is significantly changed in many cancers (Supplementary Fig. S1). For example, PCIF1 expression is increased in gastric, colorectal and liver cancers, whereas it is decreased in breast, prostate and thyroid cancers. Considering the differences of PCIF1 target genes may be involved in different cancer progression, it is possible that PCIF1 has individual function in different cancers. In fact, m^6^A modification has been documented to play opposite roles in different cancers^21^. For instance, m^6^A modification and its writer METTL3 (methyltransferase-like 3) facilitate liver cancer progression^22^, whereas they inhibit the tumorigenesis of glioblastoma stem cells^23,24^. In leukemia, m^6^A modification even plays both oncogenic and tumor-suppressive roles depending on different biological contexts^25–28^. It is interesting to investigate whether PCIF1 and m^6^Am modification have different functions in different cancers.

TM9SF1 has been previously identified as an autophagy-related gene since it was cloned in 1997^29,30^. Recent bioinformatics studies show that TM9SF1 is a tumor-associated antigen of breast cancer and a prognostic marker for cervical cancer^31,32^. In addition, TM9SF1 has been found to be increased in urinary bladder cancer by microarray analysis^33^. TM9SF1 has also been reported to interact with EBAG9 (estrogen receptor binding site associated antigen 9) and be involved in epithelial–mesenchymal transition process of prostate cancer cells^34^. In our study, we discover that *TM9SF1* is a previously undescribed tumor suppressor gene in gastric cancer. The reduction of TM9SF1 protein in gastric cancer tissues predicts an unfavorable outcome for patients. Ectopic expression of TM9SF1 attenuates the aggressiveness of gastric cancer cells. Furthermore, knockdown of TM9SF1 reverses the decreased malignancy induced by PCIF1 deletion in gastric cancer cells. Future studies will be clearly needed to determine how TM9SF1 influences the behaviors of gastric cancer cells.

## Materials and methods

### Cell culture

AGS, HEK-293T, and Hela cells were purchased from the Cell bank of Chinese Academy of Sciences (Shanghai, China). AGS cells were grown in F-12 medium with 10% fetal bovine serum (FBS, ExCell) and 1% penicillin/streptomycin (Gibco, 15140). HEK-293T and Hela cells were maintained in DMEM medium with 10% FBS and 1% penicillin/streptomycin. BGC-823 cells were obtained from the Institute of Biochemistry and Cell Biology, Chinese Academy of Sciences^35^ (Shanghai, China) and grown in RPMI-1640 with 10% FBS and antibiotics. PDX-GCSR1 cells were a gift from Department of Surgical Oncology, The First Affiliated Hospital, Zhejiang University School of Medicine^19^ (Hangzhou, China) and grown in RPMI-1640 with 10% FBS and antibiotics.

### PDX-derived gastric cancer cells

Surgical tumor specimen from gastric cancer patient with metastasis was divided into small pieces (2−3 mm) and transplanted into SCID mice for 2 months. Once tumors were grown, xenografts were collected and cut into small pieces. PDX-derived tumor cells were isolated after digesting with Type I collagenase (GIBCO, 17100017) for 30 min at 37 °C. The cell mixture was sieved and washed twice with sterile PBS at 1000 rpm for 5 min. Cells were maintained in RPMI-1640 with 10% FBS and antibiotics.

### Human specimens

Clinical samples for the m^6^Am analysis in this study were obtained from patients with informed consent in the First Affiliated Hospital, Zhejiang University School of Medicine (Hangzhou, China). The cohort Zhejiang samples were obtained from Zhejiang Cancer Hospital (Hangzhou, China). The cohort tissue array was obtained from the Sir Run Run Shaw Hospital, Zhejiang University School of Medicine (Hangzhou, China). The study was permitted by the ethics committee of Zhejiang University School of Medicine.

### Mice

All procedures involved in mice were approved by the Institutional Animal Care and Use Committee of Zhejiang University. BALB/c Nude mice, SCID mice or SCID Beige mice (6–8 weeks old) bred in specific pathogen-free facilities were used for the indicated studies.

### Animal models

The transfected BGC-823 or PDX-derived cells were subjected to following animal assays. For subcutaneous implantation assay, 5 × 10^6^ cells were resuspended with a 100 μl mixture of Matrigel (BD) and PBS with a ratio of 1:1, and then subcutaneously injected into BALB/C Nude mice. Tumor volumes were measured every week using a Vernier caliper and calculated using the following formula: volume (cm^3^) = *L* × *W*^2^/2 (*L* and *W* representing the largest and smallest diameters, respectively). For lung metastasis assays, 1 × 10^6^ cells were resuspended with 100 μl PBS and injected into SCID Beige mice through tail vein. The weight of mice was measured every week after injection. Obvious weight loss in mice was used as an end point for experiments.

### m^6^A-seq

The m^6^A-seq was performed as previously reported with several modifications^36^. Thirty microliter protein A magnetic beads (Thermo, 10002D) and 30 μl protein G magnetic beads (Thermo, 10004D) were mixed and washed twice with IP buffer (10 mM Tris-HCl, pH 7.4, 150 mM NaCl, 0.1% NP-40) and resuspended with 500 μl IP buffer. Then, 6 μg anti-m^6^A polyclonal antibody (Millipore, ABE572) was added to the beads and incubated at 4 °C overnight. Three μg total RNA was fragmented to ~150 nt using magnesium RNA fragmentation buffer (NEB, E6150S), and ethanol precipitated. The fragmented RNA was denatured at 65 °C for 5 min and chilled on ice immediately, and then incubated with the magnetic beads at 4 °C for 3 h. The magnetic beads were washed twice with IP buffer, twice with low salt buffer (10 mM Tris-HCl, pH 7.4, 50 mM NaCl, and 0.1% NP-40), and twice with high salt buffer (10 mM Tris-HCl, pH 7.4, 500 mM NaCl, and 0.1% NP-40). After washing, RNA was eluted from the beads with 6.7 mM N6-methyladenosine (Sigma, M2780) in IP buffer and purified by phenol chloroform extraction and ethanol precipitation. Fragmented total RNA (as “Input”) and immunoprecipitated RNA (as “IP”) were subjected to library construction using SMARTer Stranded Total RNA-Seq Kit v2-Pico Input Mammalian (Takara-Clontech, 634413) according to the manufacturer’s instructions. The libraries were sequenced on Illumina Hiseq ×10 with paired-end 2 × 150 bp read length.

### Quantification of m^6^Am levels by LC-MS/MS analysis

One hundred and fifty nanograms isolated RNA was decapped with 10 U RppH (NEB, M0356S) in ThermoPol buffer at 37 °C for 3 h. The decapped RNA was purified by ethanol precipitation, and then subjected to digestion with 0.5 U nuclease P1 (Sigma, N8630) in 20 μl buffer containing 10 mM ammonium acetate (pH 5.3) at 42 °C for 6 h. Next, 2.5 μl 0.5 M MES (2-(N-morpholino) ethanesulfonic acid) buffer (pH 6.5) and 1 U rSAP (NEB, M0371S) were added. The mixture was further incubated at 37 °C for 6 h and diluted to 40 μl. Five microliter of the solution was injected into LC-MS/MS analysis. The nucleosides were separated by ultra-performance liquid chromatography with a C18 column, and then detected by triple-quadrupole mass spectrometer (AB SCIEX QTRAP 6500) in the positive ion multiple reaction-monitoring mode (MRM). The mass transitions of *m*/*z* 296.0 to 150.1 (m^6^Am), *m*/*z* 268.0 to 136.0 (A) and *m*/*z* 245.0 to 113.1 (U), were monitored and recorded. Concentrations of nucleosides in RNA samples were deduced by fitting the signal intensities into the stand curves.

### Polysome profile analysis

Cells were transfected with control or PCIF1 siRNA oligonucleotides 48 h before assay. 6 × 10^6^ cells were treated with cycloheximide (Sigma, final concentration 100 μg/ml) for 5 min at 37 °C in fresh culture medium, and then washed twice with ice-cold PBS containing cycloheximide (100 μg/ml). Six hundred microliter of polysome lysis buffer (100 mM KCl, 15 mM Tris-HCl, 5 mM MgCl_2_, 1% Triton X-100, 2 mM DTT, 1 mg/ml sodium heparin, cycloheximide added freshly with final concentration at 100 μg/ml) were added to cells, and incubated on ice for 5 min. Cell lysates were centrifuged at 13,000 rpm at 4 °C for 15 min. The supernatant was added to a sucrose density gradient of 5–50% and centrifuged at 4 °C at 35,000 rpm for 190 min (Optima-L80XP, Beckman). The gradients were collected into 12 fractions using ISCO fractionator (Brandel) at 254 nm of RNA absorbance. Each fraction of RNA was extracted using TRIzol reagent (Invitrogen) and the polysome profile was analyzed by quantitative RT-PCR. The distribution of *TM9SF1* mRNA across the polysome profile was presented as the percentage. The control or PCIF1 siRNA oligonucleotides used are available in the following methods.

### Preparation of EGFP mRNAs

EGFP DNA template was amplified from pEGFP-N3 (Clontech) and the primers used were listed in the following table. The purified DNA product was then used as templates for in vitro transcription with MAXIscript™ T7 Transcription Kit (Thermo, AM1312). EGFP mRNAs starting with m^7^G-Am or m^7^G-m^6^Am were synthesized in vitro by addition of cap analogs [m^7^GpppAmG CleanCap Reagent AG (TriLink, N-7113) and m^7^Gpppm^6^AmG CleanCap Reagent AG (TriLink, N-7102)] to the in vitro transcription reaction and the level of GTP was reduced to 50 μM. After incubation at 37 °C for 1 h, 1 μl TURBO DNase 1 was added and incubated at 37 °C for 15 min. The transcripts were then purified by MEGAclear™ Transcription Clean-Up Kit (Invitrogen, AM1908).

### EGFP reporter assay

The transfection of *EGFP* mRNA into HeLa cells was performed as previously reported^9^. HeLa cells were seeded in a 6-well plate to ~30% confluence. Two hundred ng of *EGFP* mRNA starting with m^7^G-Am or m^7^G-m^6^Am were transfected into cells with 1 μl lipofectamin 2000 (Invitrogen, 11668030) according to manufacturer’s instructions. Cells were fixed and stained with DAPI, and then imaged in an ImageXpress® Micro XL Widefield High-Content Analysis System with a Nikon 20×/0.45 NA Plan Fluor ELWD objective (DAPI channel exposure: 50 ms, GFP channel exposure: 500 ms). Meanwhile, to detect the amount of EGFP mRNA transfected into the cells, total RNA was extracted from another paired transfected cells using TRIzol reagent and quantified by qPCR using SYBR GREEN mix (Takara) in Roche Light cycler 96 real-time PCR system.

### Reads pre-processing and alignment

For RNA-seq and m^6^A-seq strand orientation of the original RNA was preserved during the process of library construction. Raw sequencing reads were subjected to Trim galore (http://www.bioinformatics.babraham.ac.uk/projects/trim_galore/) for quality control and adapter trimming. The minimum quality threshold was set to 20, and the minimum length required for reads after trimming was 30 nt. All reads that were mapped to human rRNA by TopHat2 (version 2.0.13)^36^ were removed. Processed reads were mapped to human genome (hg19, UCSC Genome Browser and mm10, UCSC Genome Browser) using HISAT2 (version 2.1.0)^37^ with default parameters, and separated by strand with in-house scripts.

### Analysis of RNA-seq data

Adapter-clean reads were mapped to human genome (hg19, UCSC Genome Browser) using HISAT2 (version 2.1.0) with default parameters. The expression of transcripts was quantified as FPKM by Cufflinks (version 2.2.1)^38^.

### Identification of putative m^6^Am peak and peak intensity

For genome-base peak caller MACS2 (version 2.1.1)^39^, the effective genome size was set to 2.7 × 10^9^, under the option of -*nomodel* and *P*-value cutoff 0.01, and the number of reads in all input bam files were normalized to the same. Peak annotated by annotatePeaks.pl (Homer version 4.8)^40^, and peaks reads coverage were showed by IGV (version 2.4.15)^41^. An m^6^Am peak was identified when a peak region with > 1 RPKM_IP_ and RPKM_Input_ contains an adenosine at the transcription start site (TSS) and peak region, and the peak intensity reduced >30% after PCIF1 knockdown. Peak intensity for a corresponding region was calculated as (RPKM_IP_/RPKM_Input_).

### Motif discovery

For the analysis of sequence consensus, all peaks were chosen for de novo motif analysis with MEME (version 4.12.0)^42^, with 30 nt long peak summit centered sense sequences as input.

### Identification of PCIF1 targets

Peak intensity of each gene was calculated for both IP and input sample. Gene meeting the following conditions was defined as a potential PCIF1 target: a) gene contained a m^6^Am peak; b) gene with expression > 1 RPKM; c) gene with −0.5 < log_2_ RPKM_shPCIF1/shCtl_ < 0.5; d) gene with log_2_ peak intensity_shCtl/shPCIF1_ > 1.3. Based on these criteria, 446 high confident PCIF1-dependent m^6^Am-marked genes were identified in AGS cells.

### Migration and invasion assays

Cells stably transfected with the indicated plasmids were subjected to following experiments. For migration assays, control or PCIF1 knockdown cells (1 × 10^6^ cells in 200 μl) were resuspended in culture medium containing 1% FBS and seeded in the upper chamber of Transwell (Corning, 8 μm pore). The lower chambers were added with 800 μl culture media contained 10% FBS. After migration for 12 h (AGS cells) or 20 h (BGC-823 cells) at 37 °C, the migrated cells were stained with 0.1% crystal violet for 15 min. Cells were then photographed using an optical microscope with a photographic system (E600, Nikon, Japan) and quantified by counting in five random × 10 fields. For invasion assays, the upper chambers of Transwell were pre-coated with a 50 μl mixture of Matrigel (BD) and RPMI-1640 with a ratio of 1:9 for 2 h at 37 °C. The following procedure of invasion assays was the same as that of migration assay. Each experiment was analyzed in triplicate.

### Cell proliferation and colony formation assays

AGS or BGC-823 cells stably transfected with the indicated plasmids were subjected to following experiments. For cell proliferation assays, 2500 cells/well were seeded in 96-well culture plates. After different culture times, cells were incubated with 3-(4,5-dimethylthiazol-2-yl)-2,5-diphenyltetrazolium bromide (MTT) (5 mg/ml, 20 μl/well) for 4 h at 37 °C. After discarding the supernatant, 150 μl of dimethyl sulfoxide (DMSO) were added into each well and resuspended crystals. The absorbance of samples was measured with a spectrophotometer at 490 nm. For colony formation assays, the transfected cells were seeded in 6-well plate with 2000 cells/well and allowed to grow in 37 °C incubator for 5 days. Cell colonies were stained with 0.1% of crystal violet and imaged. Each experiment was analyzed in triplicate.

### *TM9SF1* mRNA stability

RNA was extracted from transfected cells treated with actinomycin D (MCE, HY-17559, China, 10 μg/ml) at different time points. The expression level of *TM9SF1* mRNA with different treatments was detected by quantitative RT-PCR. The mRNA level at the beginning was used for normalization.

### m^6^A-RIP qPCR

RNA immunoprecipitation (RIP)-qPCR was conducted according to previously described^43^. Briefly, the total RNA was isolated by TRIzol Reagent (invitrogen). Then isolation of mRNAs from total RNA using the magnetic mRNA isolation kit (NEB, S1550). Two microgram m^6^A antibody (ABclonal, A17924) was incubated with protein A/G magnetic beads (MCE, HY-K0202) in IP buffer (150 mM NaCl, 10 mM Tris-HCl, pH 7.4, 0.1% NP-40 in nuclease free H_2_O) at room temperature for 30 min. After saving 1/10 of the mRNAs as input, the remaining mRNAs (~2 μg) were used for immunoprecipitation in 500 μl of IP buffer added with 200 U RNase inhibitor (ABclonal, RK21401) at 4 °C for 2 h. Eluted twice with 100 μl elution buffer (150 mM NaCl, 10 mM Tris-HCl, pH 7.4, 0.1% NP-40, 200 U RNase inhibitor, 6.7 mM m^6^A) at 4 °C for 2 h. The eluted mRNAs were recovered by ethanol precipitation. Then the m^6^A-IP-mRNAs and input mRNAs were used as templates for qRT-PCR separately.

### Immunohistochemistry and tissue array

For the immunohistochemistry, the lung tissues of mice were fixed in formalin immediately after dissection. The fixed tissues were then dehydrated in ethanol and embedded with paraffin. The treated tissues were sectioned and stained with hematoxylin and eosin solution (H&E). The sections were scanned with a digital slide scanner (KF-PRO-005-EX) and analyzed by the K-viewer software. For the tissue array immunostaining, the gastric cancer tissue array was obtained from the Sir Run Run Shaw Hospital, Zhejiang University School of Medicine (Hangzhou, China), which contains 140 gastric cancer samples. The tissue array was stained using anti-PCIF1 antibody (Abcam, 1:100) or anti-TM9SF1 antibody (Bioss, 1:100), and then incubated for 1 h. The HRP-linked secondary antibody (Invitrogen) was incubated for 30 min and then colored by DAB kit (Invitrogen). The tissue array was counterstained with hematoxylin and scanned with digital slide scanner (Pannoramic MIDI, 3D HISTECH). The expression levels of PCIF1 and TM9SF1 were determined by *H*-score of the staining signal. *H*-score was acquired according to the formula: *H*-score = (percentage of cells of weak intensity × 1) + (percentage of cells of moderate intensity × 2) + (percentage of cells of strong intensity × 3).

### Immunoblotting

An equal number of cells were lysed in ice-cold RIPA lysis buffer (Beyotime Biotechnology, Shanghai, China) containing a cocktail of protease inhibitors (Roche, Basel, Switzerland). Then the samples were subjected to western blot analyses. The following antibodies were used for immunoblotting following manufacturer suggested protocols: anti-PCIF1 (Abcam, Ab205016, 1:1000 dilution); anti-TM9SF1 (Bioss, Bs-10764R, 1:1000 dilution); anti-MTSS1L (Proteintech, 27832-1-AP, 1:1000 dilution); anti-SMARCD3 (Proteintech, 12838-1-AP, 1:1000 dilution); anti-C1orf35 (Proteintech, 27930-1-AP, 1:1000 dilution); anti-GTPBP3 (Proteintech, 10764-1-AP, 1:500 dilution); anti-PIDD1 (ABclonal, A4831, 1:500 dilution); anti-PRKCE (ABclonal, A2110, 1:1000 dilution); anti-COPS8 (ABclonal, A12745, 1:1000 dilution); anti-ASH2L (ABclonal, A14543, 1:1000 dilution); anti-DNTTIP1 (ABclonal, A15558, 1:200 dilution); anti-β-actin (Sigma, A1978, 1:3000 dilution) and anti-rabbit HRP (CST, 7074, 1:3000 dilution).

### Plasmid construction

Full-length human *PCIF1* (NM_022104.3) and *TM9SF1* (NM_006405.7) were amplified from cDNA of HEK-293T cells, and cloned into pcDNA3.1 vector (Invitrogen, V79020) for transient expression and pLVX-Puro vector (Clontech, 632164) for stable expression. The following primers were used:

**Table:** 

Primer names	Sequences (5′-3′)
pcDNA3.1-*PCIF1-FL* Forward	CTTGGTACCGAGCTCGGATCCATGGCCAATGAGAATCACGG
pcDNA3.1-*PCIF1-FL* Reverse	TGCTGGATATCTGCAGAATTCTTAAGTGGGGTGAGGCTCGC
pcDNA3.1-*TM9SF1-FL* Forward	CTTGGTACCGAGCTCGGATCCATGACAGTCGTAGGGAACCC
pcDNA3.1-*TM9SF1-FL* Reverse	TGCTGGATATCTGCAGAATTCTCAGTCCATCTTGAGGTTAAC
pLVX-*TM9SF1-FL* Forward	TCGAGCTCAAGCTTCGAATTCATGACAGTCGTAGGGAACCC
pLVX-*TM9SF1-FL* Reverse	TTATCTAGAGTCGCGGGATCCTCAGTCCATCTTGAGGTTAAC
pLVX- *PCIF1-FL* Forward	TCGAGCTCAAGCTTCGAATTCATGGCCAATGAGAATCACGG
pLVX- *PCIF1-FL* Reverse	TTATCTAGAGTCGCGGGATCCTTAAGTGGGGTGAGGCTCGC
pLKO.1-sh*PCIF1#1* Forward	CCGGACGACATTCCTATCAGGTTATCTCGAGATAACCTGATAGGAATGTCGTTTTTG
pLKO.1-sh*PCIF1#1* Reverse	AATTCAAAAAACGACATTCCTATCAGGTTATCTCGAGATAACCTGATAGGAATGTCGT
pLKO.1-sh*PCIF1#2* Forward	CCGGGGTTATCCCGAATCAAGTTCCTCTCGAGAGGAACTTGATTCGGGATAACCTTTTTG
pLKO.1-sh*PCIF1#2* Reverse	AATTCAAAAAGGTTATCCCGAATCAAGTTCCTCTCGAGAGGAACTTGATTCGGGATAACC
pLKO.1-sh*TM9SF1#1* Forward	CCGGCCTCGAACACTGGAAATCCATCTCGAGATGGATTTCCAGTGTTCGAGGTTTTTG
pLKO.1-sh*TM9SF1#1* Reverse	AATTCAAAAACCTCGAACACTGGAAATCCATCTCGAGATGGATTTCCAGTGTTCGAGG
pLKO.1-sh*TM9SF1#2* Forward	CCGGCCTGAGAAGATACGTCACAAATCTCGAGATTTGTGACGTATCTTCTCAGGTTTTTG
pLKO.1-sh*TM9SF1#2* Reverse	AATTCAAAAACCTGAGAAGATACGTCACAAATCTCGAGATTTGTGACGTATCTTCTCAGG
*EGFP*-Forward	TAATACGACTCACTATAAGGACTCAGATCTCGAGCTC
*EGFP*- Reverse	TTTTTTTTTTTTTTTTTTTTTTTTCGCCTTAAGATACATTGATGAG

### Lentivirus production and infection

The pLKO.1 lentiviral vector was co-transfected with packaging plasmids Delta 8.91 and VSV-G (Addgene, 8454) into HEK-293T cells. After 48 h, the Lentivirus Concentration Kit (Genomeditech, Shanghai, China, GM-040801) was used to obtain lentivirus precipitation. Before infecting target cells, culture medium containing 10% FBS were used to resuspend viral precipitation. After infection of 48 h, cells were selected with puromycin for 4–5 days for further analyses.

### Quantitative RT-PCR analysis

RNA was isolated by using TRIzol Reagent (invitrogen). cDNA was generated by HiScript II Reverse Transcriptase (Vazyme) for RT-PCR. The LightCycler 480 II system (Roche) or CFX-96 (Bio-Rad) system was used to perform quantitative real-time PCR using ChamQ Universal SYBR qPCR Master Mix (Vazyme). The following human-specific primers were used:

**Table:** 

Primer Names	Sequences (5′-3′)
*PCIF1* Forward	CTCCGGGCAGCTGCTGAT
*PCIF1* Reverse	AAGGAAAGGCCTGCAGGAAG
*TM9SF1* Forward	ATGGACTGAGTTCTGTATG
*TM9SF1* Reverse	CTGGTAGGAGAGTTCAAC
*TM9SF1*-IP-Forward	CGCTTCCAGTCTGCTGCC
*TM9SF1*-IP-Reverse	ATTCGGTCCCCATCCAGC
Pre-*TM9SF1* Forward	GAGAGCGGCTAATCATAGGCA
Pre-*TM9SF1* Reverse	TCCCATGTTGCTGGGTCATT
*GAPDH* Forward	GAAGGTCGGAGTCAACGG
*GAPDH* Reverse	TGGAAGATGGTGATGGGAT
*EGFP* Forward	CAAGATCCGCCACAACATCG
*EGFP* Reverse	GACTGGGTGCTCAGGTAGTG
28S Forward	ACGGACCAAGGAGTCTAACA
28S Reverse	GCCTTCACCTTCATTGCGC

**Sequences for siRNA**

**Table:** 

Oligonucleotide names	Sequences (5′-3′)
siCtl	UUCUCCGAACGUGUCACGUTT
si*PCIF1*-1	CCCUACUACUUCAACCGAUTT
si*PCIF1*-2	CCUUCCAUGUUUCGUGAAATT

### Statistics analysis

GraphPad Prism 8 software was used to perform statistical analysis. Statistical significance was determined using Student’s *t*-tests, Mann–Whitney tests and the log-rank tests as indicated. Results are presented as means ± SD, and *P* < 0.05 indicates statistical significance. The western blot band intensity was quantified by Image J software.

## Supplementary information

Supplementary Information Supplementary Information

## Data Availability

The raw sequence data reported in this paper (the accession number CRA002770) was deposited in the Genome Sequence Archive in BIG Data Center, Beijing Institute of Genomics (BIG), Chinese Academy of Sciences (http://bigd.big.ac.cn/gxdsa).
