# Supplementary material for: m6Am methyltransferase PCIF1 is essential for aggressiveness of gastric cancer cells by inhibiting TM9SF1 mRNA translation
Source: Cell Discov. 2022 May 21;8:48. doi: 10.1038/s41421-022-00395-1 (PMC9124189; doi:10.1038/s41421-022-00395-1)
Supplement: Supplementary file 1 — Supplementary Information [file 41421_2022_395_MOESM1_ESM.pdf]

Supplementary Information

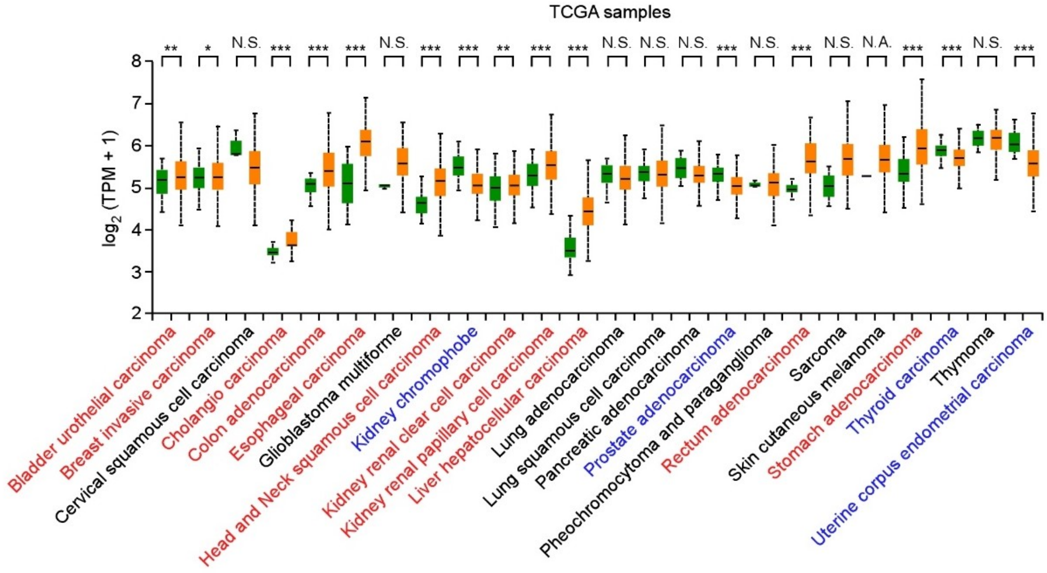

Supplementary Fig. S1 The expression pattern of *PCIF1* mRNA in main human cancers. *PCIF1* mRNA levels were analyzed in various human cancers by using TCGA database. The yellow boxes indicate tumor tissues and the green boxes represent the corresponding non-tumor tissues. *PCIF1* mRNA was significantly increased in tumor tissues with red characters compared with their corresponding non-tumor tissues. The expression of *PCIF1* mRNA was significantly decreased in tumor tissues with blue characters compared with their corresponding non-tumor tissues. Data are shown as mean ± SD. N.S. not significant; N.A. not applicable; * $P < 0.05$; ** $P < 0.01$; *** $P < 0.001$, Mann-Whitney test.

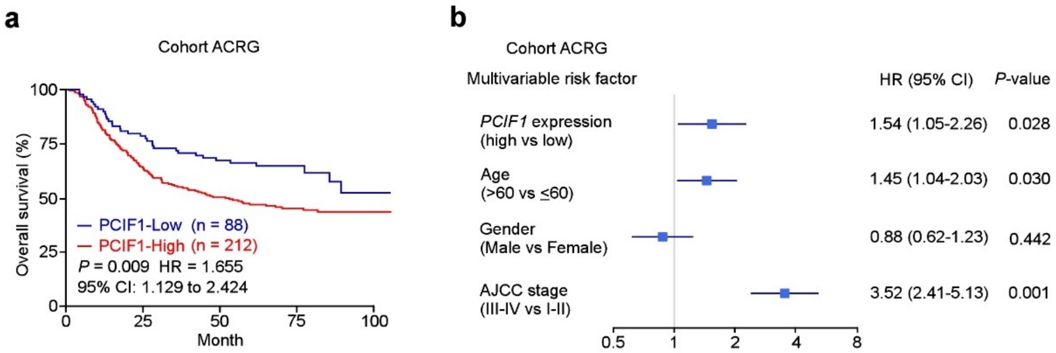

Supplementary Fig. S2 Elevated *PCIF1* mRNA is significantly associated with poor prognosis of gastric cancer patients. **a** Kaplan-Meier survival curves of *PCIF1* mRNA expression in gastric cancer tissues from cohort ACRG (GSE62254) with best cutoff. **b** Multivariable risk factor analyses of cohort ACRG. All the bars correspond to 95% CI. HR, hazard rate.

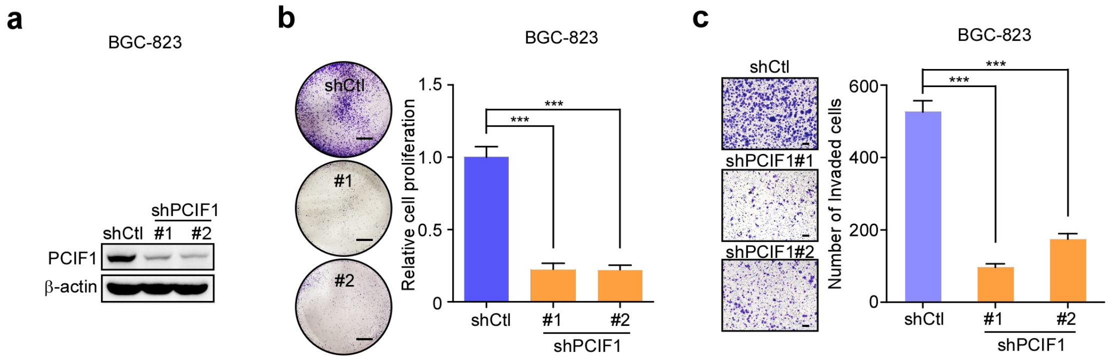

Supplementary Fig. S3 PCIF1 depletion reduces gastric cancer cell proliferation and invasion. a Western analysis of PCIF1 knockdown in BGC-823 cells. **b** The proliferation of BGC-823 cells with lentivirus-based control or PCIF1 shRNAs. Representative images of cell colonies are presented. Cell colonies were also counted. Scale bars, 5 mm. **c** Migration analysis of BGC-823 cells with lentivirus-based control or PCIF1 shRNAs. Representative images of invaded cells and quantification of invaded cells are shown. Scale bars, 100 µm. Data are shown as mean ± SD. *** $P < 0.001$, Student's *t*-test.

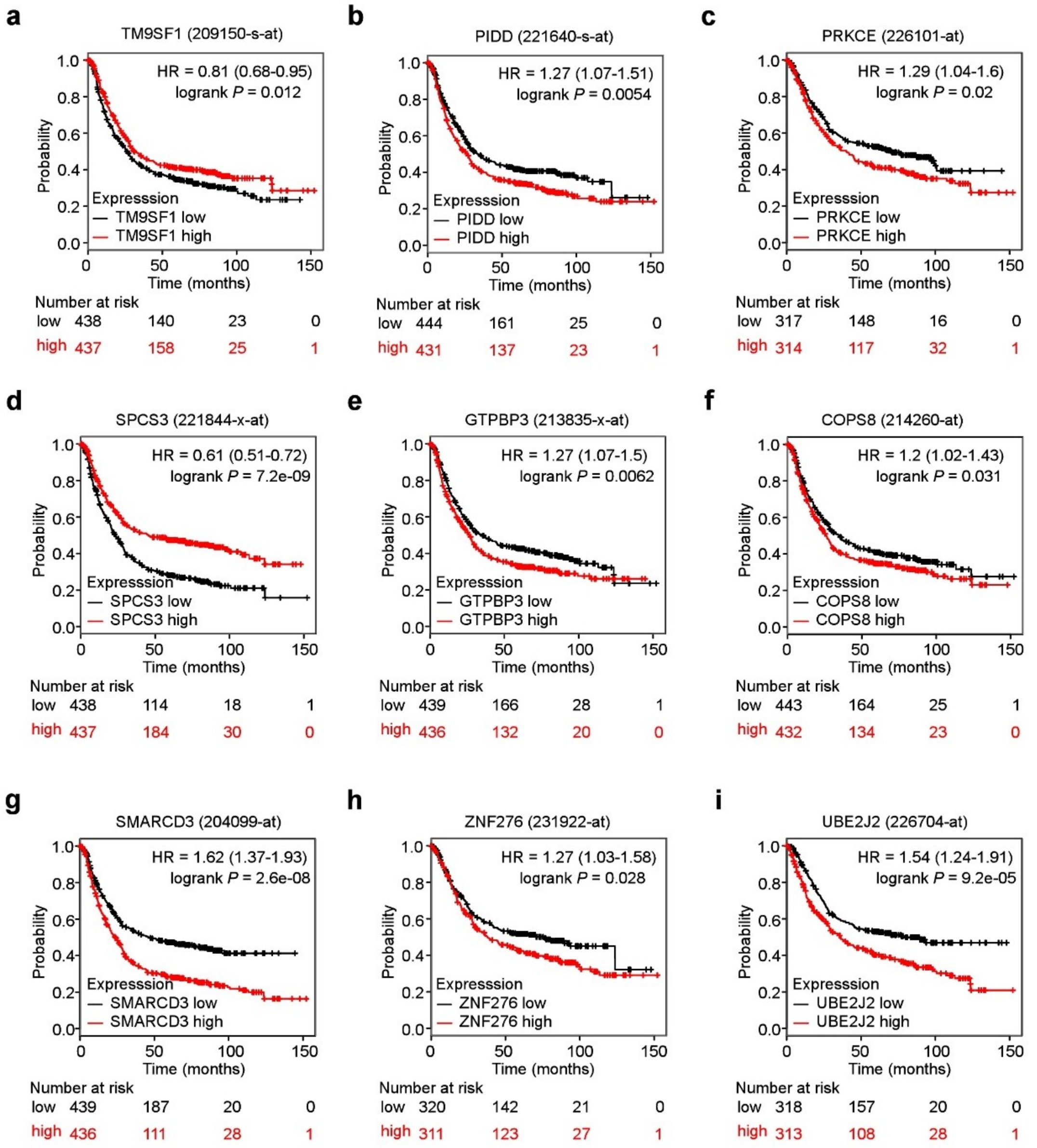

Supplementary Fig. S4 Kaplan-Meier survival curves of the indicated genes in gastric cancer patients. a-i Top 20 downregulated genes in m^{6}Am abundance in PCIF1-knockdown AGS cells were subjected to Kaplan-Meier Plotter analysis with median cutoff by the log-rank test. Nine top genes that are significantly associated with the overall survival of gastric cancer patients are shown.

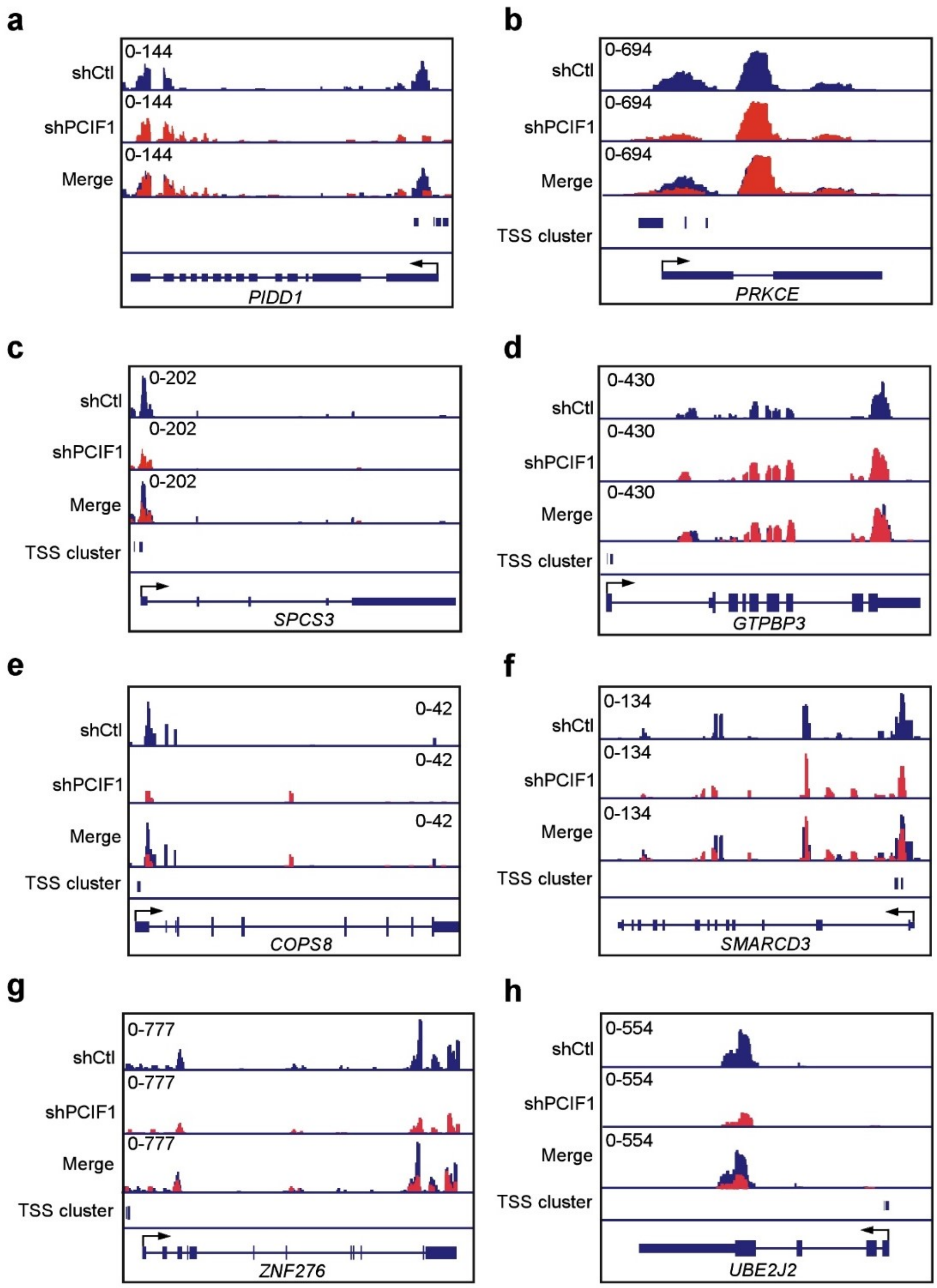

Supplementary Fig. S5 The m^{6}A/m^{6}Am peaks analysis of the indicated genes. a-h The m^{6}A/m^{6}Am peaks on the indicated genes in control or PCIF1-depleted AGS cells are shown.

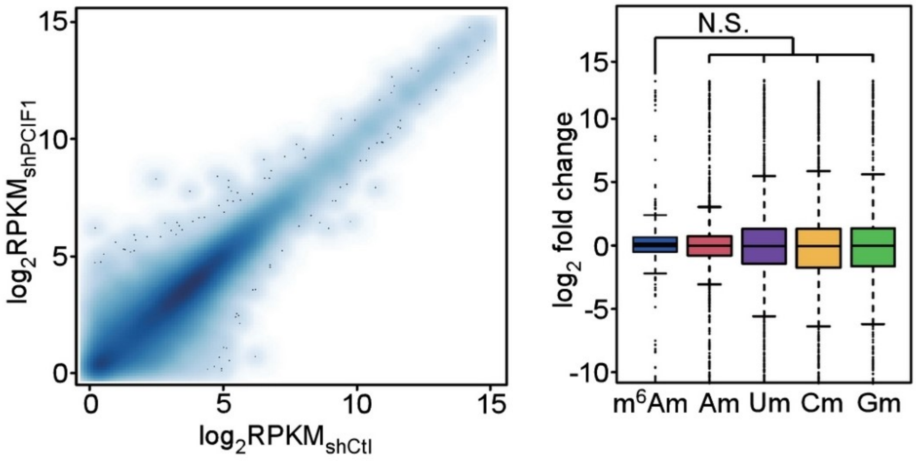

Supplementary Fig. S6 The expression of m^{6}Am-containing transcripts in control and PCIF1-depleted AGS cells. The fold change of transcripts classified in five groups (m^{6}Am, Am, Um, Cm, and Gm) based on the first nucleotides were analyzed. N.S. not significant, Student's *t*-test.

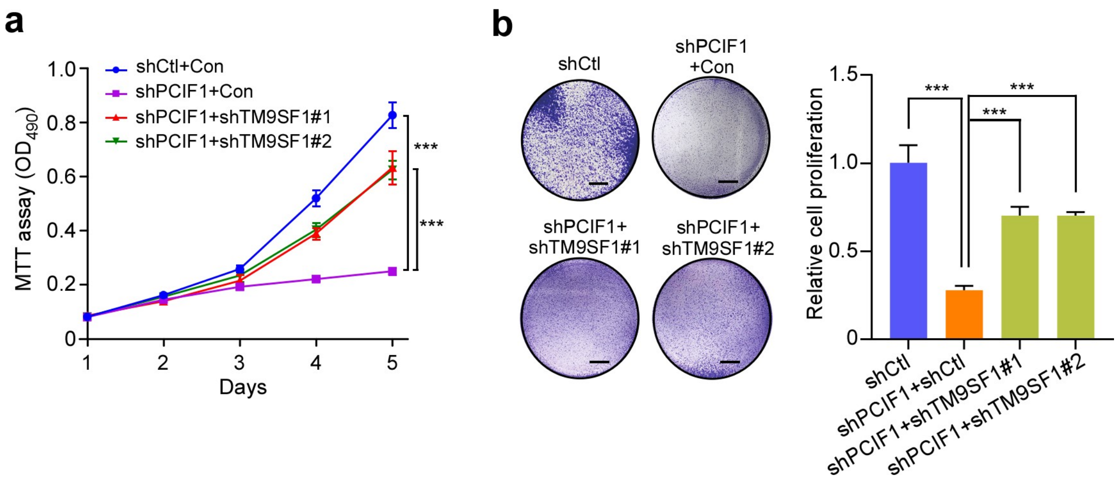

Supplementary Fig. S7 Depletion of TM9SF1 rescues the inhibition of cell proliferation induced by PCIF1 knockdown. a, **b** BGC-823 cells transfected with the indicated shRNAs were subjected to MTT and colony formation assays. Representative images of cell colonies are shown (**b left**). Cell colonies were counted and normalized to controls (**b right**). Data are shown as mean ± SD. Scale bars, 5 mm. *** $P < 0.001$, Student's *t*-test.

Supplementary Table S1. The high confident m^{6}Am-marked genes of AGS cells depleted of PCIF1.

CHR	Star	End	Gene	Ctrl_IP1_ Peak_RPKM	Ctrl_IP2_ Peak_RPKM	Ctrl_Input1_ Peak_RPKM	Ctrl_Input2_ Peak_RPKM	KD_IP1_ Peak_RPKM	KD_IP2_ Peak_RPKM	KD_Input1_ Peak_RPKM	KD_Input2_ Peak_RPKM
chr1	109793934	109794220	CELSR2	114.085	94.9947	5.27534	2.02081	57.0508	61.6934	5.16517	11.1318
chr1	1169877	1170387	B3GALT6	191.161	180.409	10.0583	10.4825	52.0617	74.6085	8.39999	11.296
chr1	1209930	1210280	UBE2J2	15.3501	26.0081	0	0.825646	15.257	10.0825	1.26621	1.29947
chr1	121260616	121260824	EMBP1	36.3945	15.3124	1.66426	0	8.36307	3.93657	0	0
chr1	149288128	149288503	LOC100996741	40.8838	29.5025	1.60933	0.770603	20.1732	27.4126	10.2422	2.42567
chr1	149833288	149833489	HIST2H4B	17.6021	34.8367	7.50621	5.75077	9.59362	11.4499	5.14462	3.77125
chr1	152284953	152285223	FLG-AS1	115.507	95.4372	11.7347	5.88655	30.2158	28.4128	8.75401	14.5989
chr1	156697687	156697914	ISG20L2	181.629	81.6335	25.9244	27.664	66.8778	73.5844	32.7013	38.4055
chr1	161016357	161016557	USF1	40.6217	35.0109	7.54374	0.72244	2.96664	18.4115	1.47724	4.54813
chr1	161128389	161128605	USP21	100.098	51.2197	18.1608	12.0407	35.7096	39.7779	11.6264	22.4599
chr1	161719319	161719597	DUSP12	60.7822	43.2233	1.24521	1.78334	21.616	5.8907	3.1049	1.28
chr1	165877111	165877331	MIR3658	272.201	229.162	30.8607	72.244	171.93	102.518	61.7755	82.6933
chr1	168105410	168105598	GPR161	34.514	66.9955	5.52394	3.51609	47.105	27.8743	11.9374	1.89276
chr1	173684129	173684359	KLHL20	44.4389	42.622	1.96793	0.628209	22.5723	23.3479	5.13823	3.29575
chr1	173684550	173684756	KLHL20	47.0718	25.1535	2.1972	1.4028	23.7619	51.3912	5.73685	7.35944
chr1	17766510	17766657	RCC2	23.1767	23.817	5.13179	0	1.00906	0	0	0
chr1	185286106	185286339	IVNS1ABP	19.1214	27.0471	0	0	4.45633	0	1.90203	0
chr1	203767981	203768279	ZBED6	295.934	280.557	32.4026	24.243	188.651	195.648	32.2217	39.173
chr1	204246103	204246366	GOLT1A	86.3505	85.8723	12.5041	4.39845	46.299	61.0214	10.5024	14.883
chr1	204378713	204379210	PPP1R15B	1482.9	1438.98	121.89	127.35	1610.65	1481.43	181.316	195.461
chr1	206284889	206285073	RHEX	23.5095	11.8021	0	0	0	2.67002	0	0
chr1	226736637	226736837	STUM	3.93113	5.60175	0	0	3.7083	3.06858	0.73862	0.758022
chr1	228291781	228292447	C1orf35	41.3182	34.9058	0.679616	0.867796	20.2676	7.60233	5.32339	0.910537
chr1	231473266	231473488	EXOC8	62.7185	80.8636	11.6948	5.95517	96.8774	135.73	27.2169	15.2273
chr1	236227141	236227768	NID1	84.9455	68.1143	8.28151	3.1628	32.2834	37.3491	5.50662	5.95903
chr1	244217585	244218796	ZBTB18	298.974	290.033	11.462	15.2721	206.146	164.705	11.7106	13.7708
chr1	24649168	24649415	GRHL3	41.9109	32.8848	3.66497	0	20.4182	21.1198	2.39229	4.29648
chr1	27018652	27018814	LOC101928728	4.17221	8.93653	0	0	1.95232	2.02175	0	0
chr1	28285441	28285643	XKR8	150.499	143.51	22.4071	16.4516	74.9004	66.0807	15.3574	12.7588
chr1	3375681	3375952	ARHGEF16	43.5181	40.3078	1.6702	2.66583	20.7993	53.785	5.99618	12.8668
chr1	41827587	41827941	FOXO6	38.1268	61.7142	0	0	21.3699	6.06781	0.417299	0.428261
chr1	46049064	46049406	NASP	33.5973	42.331	0	0	7.86067	12.9285	0	0
chr1	47904925	47905168	LINC01389	37.2082	30.5445	1.24177	0	31.7419	41.672	1.82375	1.87166
chr1	64239793	64240034	ROR1	128.319	152.247	6.8864	9.59257	59.7021	50.2941	4.29074	5.03251
chr1	6662261	6662513	KLHL21	178.63	157.411	7.55523	16.3944	264.191	131.269	19.8665	28.2412
chr1	68299739	68299973	GNG12	13.9998	10.1741	0	0	3.80339	0	0	5.18306

chr1	76558967	76559173	ST6GALNAC3	66.7911	40.1096	4.39441	9.81957	46.8038	55.1152	30.1185	28.7018
chr1	7832846	7833289	VAMP3	46.1442	50.264	4.76805	6.197	24.7778	23.5512	16.0062	16.4267
chr1	855856	856104	LOC100130417	48.5121	54.8732	2.09375	1.33271	4.46358	7.92394	1.3922	0
chr1	86173452	86173898	ZNHIT6	89.4126	79.8517	13.9709	7.78111	108.144	105.013	29.0301	16.7547
chr1	90286998	90287167	LRRC8D	6.39904	26.5558	0	0	1.87146	3.87601	0	0
chr1	91486354	91486584	ZNF644	169.269	107.635	45.9048	24.4292	118.26	84.0167	51.0392	30.169
chr2	10589165	10589595	LOC101929715	28.3407	29.3115	0	0.672037	14.1433	17.1269	0.343544	1.76284
chr2	113340966	113341364	CHCHD5	55.3625	43.2861	8.26278	3.32173	30.9919	18.1043	6.50625	2.23517
chr2	114257833	114257991	FOXD4L1	8.29353	16.8407	0	0	1.87762	0	1.86992	1.91904
chr2	11457870	11458293	ROCK2	41.225	40.0433	11.8662	10.5483	27.291	27.1	22.8544	26.4986
chr2	11458469	11458846	ROCK2	47.3308	34.9449	11.4777	9.20524	29.782	35.6192	29.7642	30.6758
chr2	118772009	118772462	CCDC93	35.2905	14.5299	0.99917	0.956874	10.4782	6.7739	0	0.334668
chr2	129075842	129076258	HS6ST1	59.791	67.5138	5.40886	12.712	36.1133	35.4292	9.54459	10.2646
chr2	153246162	153246362	FMNL2	41.2769	43.4135	3.01749	8.66928	45.2413	48.3301	31.7607	13.6444
chr2	163174495	163174729	IFIH1	180.817	105.795	11.0951	2.82489	152.056	129.47	12.5417	18.2482
chr2	171570034	171570232	LINC01124	19.1163	35.0962	4.3708	0	0.798677	9.09786	0	0.898583
chr2	174889840	174890090	LINC01960	29.7395	60.2251	1.38467	0	13.9162	19.6514	1.38106	0
chr2	178481413	178481817	TTC30A	21.08	26.1593	0	0	4.69717	2.4321	0.427308	0
chr2	178936988	178937246	PDE11A	17.2904	18.5173	0	1.28105	8.58114	5.07787	0.669119	0
chr2	20101066	20101424	TTC32	53.2412	60.2542	2.90084	9.69379	32.246	30.1907	9.16207	12.4245
chr2	208618381	208618726	MIR4775	185.732	174.953	12.6822	5.86328	161.661	229.031	20.5529	12.3041
chr2	219081192	219081454	ARPC2	26.8296	16.0244	0	0.630748	12.6752	7.50052	0	0
chr2	219825493	219825814	CDK5R2	72.2545	69.3674	0.470015	0	72.5487	80.2992	2.7612	5.19517
chr2	220360811	220361428	LOC100996693	10.5164	8.91626	0	0	6.15124	3.4504	0	0
chr2	222437666	222438101	EPHA4	28.3162	21.8919	0	0.332156	3.75093	5.29065	0	0.348516
chr2	222438658	222438841	EPHA4	17.1853	48.977	1.6489	0.789552	4.05279	2.51523	0	4.1422
chr2	237992885	237993357	COPS8	16.0383	14.7226	0	0	3.68542	4.51038	0.365747	1.88474
chr2	239142713	239142956	LOC643387	33.4335	31.6971	0	0	21.9751	22.7302	2.43167	1.24777
chr2	26203380	26203434	KIF3C	77.6032	230.563	0	21.4221	193.28	169.827	12.7876	32.9481
chr2	32582351	32582637	BIRC6	47.7383	73.3983	0.605187	0	22.6702	23.4763	1.81083	2.48838
chr2	32582873	32583024	BIRC6	28.6374	31.533	5.99502	3.8275	41.2579	28.4504	13.6963	14.056
chr2	45264	46062	FAM110C	19.3114	15.602	1.51828	1.86379	12.0883	14.365	3.46131	2.89844
chr2	45878453	45878743	PRKCE	84.4968	62.2953	1.04052	2.98941	50.126	59.2553	5.09393	7.31883
chr2	47143492	47143696	TTC7A	24.409	16.4757	0	0	7.9983	4.51261	12.3103	2.97264
chr2	55509703	55509905	PRORSD1P	22.7046	36.0508	0	0	8.07749	10.6337	0	0.750517
chr2	62934106	62934427	EHBP1	353.924	419.259	46.5315	39.6104	262.469	186.409	51.0821	37.3107
chr2	68384319	68384536	WDR92	71.0161	68.7166	2.39286	6.09239	70.6885	43.0157	4.77325	9.83887
chr2	69871782	69871929	AAK1	13.3712	15.2429	0	0	0	3.1312	0	0
chr2	75059861	75060061	HK2	39.878	19.5442	0.865418	0.82628	3.95345	9.00688	2.58949	2.66879
chr2	9347301	9347611	ASAP2	192.752	196.965	29.2016	30.762	196.66	155.904	41.9345	41.0799
chr2	96490053	96490686	LINC00342	279.542	295.49	59.882	53.2579	240.33	227.403	76.9074	68.863
chr2	97481182	97481391	CNNM3	56.271	47.7955	0.828151	3.95349	30.2657	7.83548	1.65199	1.70258
chr3	100120461	100120672	TOMM70	35.3988	23.23	0	0	10.5449	16.7245	0	0

chr3	101395908	101396223	ZBTB11	49.5031	30.6762	0.957935	0	14.5978	22.8926	0.93793	0.962568
chr3	11248133	11248275	HRH1	106.621	106.03	9.75118	8.14642	43.4323	66.8885	4.86289	12.5295
chr3	150127597	150128255	TSC22D2	295.532	314.353	22.0121	29.6442	219.117	191.903	25.1445	31.5651
chr3	183528297	183528543	YEATS2-AS1	95.3486	117.842	9.19968	12.3343	36.1786	53.014	22.2186	16.6395
chr3	183543894	183544114	MAP6D1	30.1082	28.9544	1.57349	0	21.5643	11.1656	2.35408	4.85235
chr3	183978386	183978634	CAMK2N2	30.5245	33.8579	0	3.99813	24.2308	36.9784	1.3922	6.45676
chr3	184428662	184428914	MAGEF1	110.504	125.239	11.6763	21.6407	92.2472	87.0795	34.9376	30.3593
chr3	48699351	48699711	CELSR3	63.8349	45.4423	2.88473	1.37713	17.5709	29.1131	2.39768	3.45955
chr3	48700254	48700669	CELSR3	24.1043	29.3032	2.91948	2.78745	22.8633	12.2328	4.99179	4.28722
chr3	49754988	49755241	AMIGO3	75.8716	53.7888	1.36825	5.87867	26.8772	9.70918	4.7764	2.10972
chr3	50395960	50396248	TMEM115	62.7889	77.3158	0.523871	1.00339	19.5716	33.0298	1.02586	4.21123
chr3	51431951	51432161	RBM15B	91.1024	89.3612	12.9321	0.688038	29.6664	21.1878	4.92413	2.16578
chr3	57742758	57743463	SLMAP	586.417	554.017	41.0893	33.4065	322.964	353.43	34.7832	40.8579
chr4	175204628	175204856	CEP44	13.2187	27.6402	0	0	22.7703	13.4587	1.29582	0
chr4	177240674	177240872	SPCS3	13.6545	10.2364	0	0	3.19471	6.61662	0.871882	0
chr4	22517906	22518239	ADGRA3	27.939	21.8687	0.906155	1.30169	7.12707	7.37196	0.443616	5.46322
chr4	2252649	2252851	MIR4800	65.5822	32.2512	8.56849	3.2724	42.2745	23.5103	13.6739	4.40395
chr4	2937407	2938131	NOP14-AS1	184.43	197.299	9.37757	10.3776	80.5173	62.0921	5.91712	5.65376
chr4	30788504	30788814	PCDH7	51.5697	42.0131	5.35362	13.9827	25.36	35.6351	12.3898	18.5838
chr4	39064286	39064525	KLHL5	393.113	288.291	139.512	133.002	323.972	220.835	266.398	194.739
chr4	491064	491296	ZNF721	82.1566	101.715	2.9842	8.54773	89.9751	40.9404	9.67338	22.2399
chr4	57253812	57254029	AASDH	27.1737	13.5526	0	0	12.9876	9.89864	0	0
chr4	6718675	6719142	BLOC1S4	115.605	85.1658	1.93843	6.49732	33.9862	34.1683	0.948976	3.89562
chr4	681738	681974	SLC49A3	43.8643	71.2087	1.2786	0	1.25705	22.7543	1.2519	3.21196
chr4	77817653	77818009	SOWAHB	142.394	111.426	1.45857	2.78521	77.2922	45.0804	8.72862	1.9991
chr4	84205028	84205296	COQ2	47.4138	35.1126	4.52084	5.54964	39.5345	26.2752	8.37397	9.95818
chr5	130695023	130695295	CDC42SE2	133.447	125.627	22.7421	35.0596	81.2555	91.9445	28.2413	41.8027
chr5	131630625	131630952	SLC22A4	46.8851	43.6834	6.92086	0.883719	35.8356	36.5977	10.3904	5.56346
chr5	1345931	1346242	CLPTM1L	118.397	129.687	2.91077	4.64592	44.3566	63.1476	4.74997	7.79958
chr5	140474413	140474685	PCDHB2	37.577	40.6745	2.77343	11.1553	26.1763	9.5893	7.60344	5.01632
chr5	140557821	140558104	PCDHB8	7.40849	19.2993	0	0	6.2897	5.42151	1.04399	0
chr5	140589331	140589750	PCDHB12	53.4784	42.4476	0	2.75872	12.7445	14.281	0.352563	2.53277
chr5	140595744	140596088	PCDHB13	54.853	51.2951	0.877179	0.840047	7.33037	21.8547	0	3.08497
chr5	140602517	140602785	PCDHB14	86.0546	90.9239	0	5.93048	11.0696	10.3049	0	5.09119
chr5	140603147	140603350	PCDHB14	92.9529	39.3226	0	0.711764	18.2675	34.0113	0	5.22774
chr5	140604288	140604691	PCDHB14	62.755	42.7429	0.374379	0.358531	13.2505	11.8022	0.366561	2.63333
chr5	140698442	140698989	TAF7	169.036	168.327	47.4635	36.5557	100.318	105.682	39.1343	53.3433
chr5	140736503	140736810	PCDHGA4	58.903	28.7386	0	0	25.6078	17.4919	0	0.493825
chr5	140739869	140740035	PCDHGB2	40.2586	25.3091	10.9066	1.74082	13.4035	23.1068	4.44952	2.73984
chr5	140755421	140755653	PCDHGA6	56.4818	47.0837	0	1.24559	19.1809	5.29065	0	0.653467
chr5	14713286	14713899	LOC100130744	41.0428	63.9678	2.46125	0.235706	16.6964	21.7754	1.20493	0
chr5	180687319	180687587	TRIM52	87.2615	68.6046	5.16667	1.84988	70.8081	80.0476	9.66227	3.98327
chr5	20304835	20305093	CDH18-AS1	50.8234	43.2071	0	1.92158	45.9704	36.1799	4.01471	6.20649

chr5	25189903	25190412	LINC02228	75.4244	74.5191	11.9016	10.3894	43.8064	42.7903	9.49649	13.2828
chr5	36875747	36876367	NIPBL-DT	67.1538	58.3757	1.675	2.13234	35.1985	26.9414	0.835319	1.7218
chr5	41925417	41925639	FBXO4	28.9227	33.4339	0	0.650847	19.3767	15.2047	4.65796	4.78032
chr5	41926131	41926357	FBXO4	22.0329	26.0258	0	0.639328	4.59436	13.5778	0	6.70816
chr5	43067067	43067307	LOC100506639	25.1156	51.9329	0.628645	2.40813	30.9025	23.0143	3.6931	5.05348
chr5	43476271	43476514	TMEM267	115.153	46.47	11.3964	2.72026	19.5232	32.3479	2.84169	25.6263
chr5	472664	473027	PP7080	398.836	407.993	10.9667	15.0233	218.257	186.769	19.0229	10.783
chr5	74151595	74151805	LOC441086	28.3234	37.227	9.89049	12.5909	14.3077	17.9358	10.6868	19.4864
chr5	74324486	74324885	GCNT4	31.5081	43.9032	0.433793	0	11.0974	13.1337	0	1.33774
chr5	78808602	78809014	HOMER1	119.759	123.337	12.6032	6.0166	74.8469	85.8555	15.5034	8.20502
chr6	105851424	105851656	PREP	31.065	44.0655	0.650322	0	13.4266	15.872	0	0.653467
chr6	109786828	109787489	MICAL1	96.5277	91.5501	3.66591	1.75006	81.3418	55.0001	6.79039	2.96084
chr6	119215420	119215719	ASF1A	24.8658	34.3773	2.3155	1.10539	28.5601	21.9079	4.04157	7.73563
chr6	13487401	13487615	GFOD1	26.9423	20.9411	0	0	10.3971	2.86783	1.3806	0
chr6	139012773	139013498	FLJ46906	167.186	151.054	7.69981	3.58729	172.679	173.11	14.6705	6.90061
chr6	147830273	147830548	SAMD5	179.641	244.949	37.3072	55.1682	172.605	157.892	53.1806	61.1931
chr6	159030712	159030903	MIR7161	76.8389	74.7877	11.0589	9.83426	97.8526	54.6239	30.937	31.7496
chr6	160147204	160147730	SOD2	72.2158	99.3586	4.27773	3.7701	28.2604	38.9167	2.29739	11.1622
chr6	3023645	3023848	LOC101927759	140.507	132.648	24.7262	19.5377	53.7514	64.5365	29.7642	50.8341
chr6	31626928	31628298	C6orf47	123.24	110.745	6.31692	8.08186	78.3765	71.7203	7.30853	10.5193
chr6	32812994	32813197	PSMB8	117.866	111.966	9.37891	13.8392	88.8066	80.6707	18.709	35.058
chr6	3747421	3747817	PXDC1	126.646	129.417	27.9731	35.8889	67.0889	73.1964	20.0533	38.6391
chr6	38607496	38607687	BTBD9	8.49297	52.2998	0	3.46086	19.8708	29.1512	6.32685	9.31516
chr6	41065264	41066222	OARD1	321.029	264.007	14.9615	11.01	148.022	154.23	18.3498	9.96981
chr6	42713375	42713537	TBCC	95.1265	124.218	11.7526	12.2412	102.497	98.0547	31.969	39.5377
chr6	43422470	43422739	DLK2	24.8436	46.3342	0	0	42.4594	37.0739	2.7458	3.38151
chr6	44226337	44226668	SLC35B2	75.962	81.7895	1.04582	0	37.7429	67.2857	3.65084	8.06282
chr6	46998924	46999125	ADGRF1	7.39789	5.04181	2.58334	0.822169	13.3749	13.8505	10.3064	6.1962
chr6	64286405	64286744	PTP4A1	710.851	706.828	84.116	99.3089	439.746	444.446	84.1024	121.641
chr6	66499028	66499218	SLC25A51P1	4.82771	3.68536	0	0	6.24556	2.42256	0.777495	0.797918
chr7	100076324	100076893	TSC22D4	69.1341	99.4829	12.7759	14.2312	58.0859	90.9466	17.2936	18.1359
chr7	122526166	122526449	CADPS2	36.7803	18.4162	0	0	47.4973	35.8771	3.66006	6.28691
chr7	128387860	128388299	CALU	154.619	112.928	10.654	11.8487	97.9871	52.4244	16.8251	12.7776
chr7	140395553	140395753	NDUFB2	9.46258	26.7828	0	0	3.95345	5.73165	0	0
chr7	148787600	148787800	ZNF786	24.3323	26.7828	0	0	22.1393	13.1009	0.863163	6.22718
chr7	149194504	149194719	ZNF746	47.1557	82.1496	4.0252	6.14906	86.0566	90.64	18.4677	23.9984
chr7	150082570	150082770	ZNF775	26.2075	26.6083	1.50875	0	5.93328	9.97288	0	0
chr7	150945615	150945842	SMARCD3	53	26.786	0	0	31.349	56.2704	0.760496	10.973
chr7	30324807	30325033	LOC105375218	253.378	388.528	30.709	33.8844	230.374	323.83	48.3698	58.361
chr7	30411093	30412080	DKFZP586I1420	76.2063	64.9848	4.12727	2.48865	53.2012	42.9041	3.74174	6.29765
chr7	32468394	32468626	PDE1C	7.57473	29.9528	0	0	2.72652	0	0	0
chr7	32919159	32919342	KBTBD2	104.155	97.3057	9.45812	12.6425	81.2294	88.5922	46.224	86.5291
chr7	5467440	5467950	LOC100129484	36.578	37.4704	4.41193	3.56435	7.75187	6.10092	4.73893	0

chr7	75157061	75157329	PMS2P3	45.3962	30.2508	3.22917	1.23325	71.9882	73.326	9.01812	7.96654
chr7	76610014	76610474	DTX2P1-UPK3 BP1-PMS2P11	146.705	146.133	8.85569	13.8206	130.919	83.7188	12.2033	23.3998
chr7	97572275	97572501	ASNS	21.5331	30.748	0	0	6.99726	7.97069	3.05544	3.93627
chr7	97582782	97583036	MIR5692A1	31.4	64.4064	0	5.20491	18.0551	19.9866	8.15587	4.90329
chr7	99949309	99949509	PVRIG2P	38.0009	49.7155	4.52624	3.6122	27.4414	82.0844	5.90896	12.8864
chr8	126011372	126011806	SQLE	266.001	203.289	9.38622	10.6535	236.169	188.074	26.5495	26.5482
chr8	128203474	128203647	CASC19	124.24	103.767	37.0179	28.6571	55.7597	79.5142	98.7898	80.218
chr8	143747260	143747718	JRK	49.8807	58.7938	1.13373	1.44328	51.1014	35.0407	6.03083	1.94235
chr8	144639121	144639475	LOC100310756	90.8835	78.9293	9.28979	2.80095	10.7212	15.7285	6.82728	0.502597
chr8	144680847	144681033	TIGD5	69.0414	81.3157	2.43346	3.88409	21.5321	40.4194	5.5595	3.26031
chr8	145320844	145321097	SCX	22.2712	22.1413	2.38537	0	1.75888	13.9481	0	1.79768
chr8	145489930	145490135	SCX	26.8468	26.6425	0.735974	0	5.78857	23.2014	0.720605	0.739534
chr8	23083377	23083641	LOC389641	69.4897	46.1508	2.28598	8.75685	26.9695	19.7598	4.47648	13.208
chr8	29931212	29931409	SARAF	442.593	462.241	57.1088	52.0095	492.877	468.84	86.7545	73.1547
chr8	33369609	33369968	TTI2	63.6361	49.1982	4.33914	1.38097	31.2752	41.5106	7.69393	14.3723
chr8	37963097	37963348	ASH2L	50.118	61.9317	0.601095	2.3026	74.4615	84.9666	9.41667	17.516
chr8	41455908	41456432	LOC102723729	404.366	399.819	13.2447	16.8202	298.363	289.582	25.0905	17.07
chr8	48649590	48650011	CEBPD	288.341	303.299	13.5671	7.85064	291.861	377.313	18.8625	25.7793
chr8	6577494	6577700	AGPAT5	43.8913	31.9517	7.32402	4.20839	25.9221	17.8752	10.7566	6.62349
chr8	68564281	68564504	CPA6	31.5217	31.8109	7.76159	5.18741	5.67312	8.81227	19.3534	5.58492
chr8	74884162	74884368	ELOC	73.4958	64.6554	19.3249	5.6155	96.7253	108.909	41.0631	31.0927
chr8	82645906	82646207	CHMP4C	36.5687	27.9157	16.5411	11.5206	8.37756	16.8211	5.88933	16.6211
chr8	8560712	8561213	CLDN23	42.8946	34.6615	0.301147	0	58.6223	52.6742	4.42287	2.11823
chr8	90769294	90769521	LOC101929709	125.652	126.277	6.09986	3.64	33.4389	62.0418	7.60496	3.13515
chr9	115513119	115513352	SNX30	29.2445	52.892	1.94259	6.82132	60.4788	63.8738	8.87612	14.3146
chr9	115652688	115652993	SLC46A2	22.3409	40.8652	0.494671	0	13.131	22.134	0.484341	0.994127
chr9	127116315	127116553	LOC100129034	127.184	66.4913	10.7768	9.10639	16.2043	21.9184	9.31033	3.82196
chr9	131707801	131708385	NUP188	133.328	159.15	3.55651	6.79134	105.335	105.996	7.9813	10.9676
chr9	131938900	131939697	IER5L	1607.74	1584.86	41.9136	46.2385	1740.51	1610.49	60.4322	69.4265
chr9	140098513	140099213	TMEM203	476.605	470.302	14.0939	12.2762	317.406	306.702	12.0843	15.7586
chr9	140195446	140195720	NRARP	356.697	433.259	39.7966	25.9343	247.019	239.665	40.953	55.194
chr9	19049769	19050127	RRAGA	546.113	487.806	38.3508	27.8482	256.059	246.429	25.9961	24.1381
chr9	21333021	21334526	KLHL9	232.904	204.316	10.8105	14.2746	167.28	137.973	10.3235	15.4867
chr9	21481048	21481643	IFNE	28.1719	25.7913	0.290897	1.11096	11.1627	6.88073	8.9943	0.598049
chr9	35748387	35748844	GBA2	298.46	268.319	19.6944	19.8886	197.586	179.528	16.9988	19.466
chr9	35906685	35906982	HRCT1	49.4149	23.1049	2.53998	0	23.4734	14.9813	5.47126	0.510453
chr9	44998680	44998896	LOC105379807	30.3328	37.6043	0.698494	1.33785	17.1681	14.2064	0.683907	2.80749
chr9	71395085	71395387	FAM122A	66.8206	41.2712	11.4905	9.09031	76.1307	64.0134	19.0769	21.5861
chr9	71586791	71587115	LOC101927069	59.2454	37.0866	7.47892	2.55025	4.88081	4.54893	0.532817	0
chr9	78506040	78506299	PCSK5	179.102	194.655	46.0197	55.7869	229.657	255.32	87.2655	109.46
chr10	101379142	101379935	SLC25A28	26.9337	24.2808	3.27396	1.04197	22.3348	26.8461	8.27243	5.16034
chr10	102748805	102749288	TWNK	78.4056	55.6695	5.93503	4.78635	42.9948	42.8838	7.03447	5.96373

chr10	103825915	103826861	HPS6	186.583	232.715	7.97435	7.33132	124.969	122.938	8.27629	10.0963
chr10	115805147	115805577	ADRB1	87.7648	69.0448	0	0	123.495	55.3057	2.74835	1.41027
chr10	120790278	120790743	NANOS1	58.0511	62.9444	5.51585	3.418	14.6737	17.8175	1.58843	1.30412
chr10	22542264	22542515	LOC100130992	20.8825	26.7813	0	0	21.8657	4.27889	4.70833	2.41601
chr10	22725088	22725270	LOC100499489	11.1412	10.3408	0	0.908	1.73778	0.899788	0	0
chr10	22726023	22726243	LOC100499489	14.7469	27.6383	0	0.751164	0	0.74437	0	0
chr10	31606910	31607581	ZEB1	91.2614	91.0488	0.257949	1.4777	44.7783	40.7573	3.60187	2.3864
chr10	51188113	51188352	FAM25E	49.893	39.2591	4.41893	1.2091	22.3429	48.1471	3.09046	3.80597
chr10	70980296	70980586	HKDC1	28.9187	28.4916	5.72283	0.498235	24.04	13.7557	8.15029	4.70496
chr10	75256385	75256544	PPP3CB	27.1965	27.3041	0.948898	0	5.59744	22.1941	0	0.953487
chr10	75561264	75561539	ZSWIM8-AS1	129.132	78.4245	4.93772	5.77952	90.0781	71.4142	6.98332	18.1925
chr10	81462061	81462253	NUTM2B	11.6023	7.29394	0	0	8.49819	2.39733	0	0
chr10	81463006	81463217	NUTM2B	6.83135	3.98228	0	0	0.702996	3.63575	0.700114	0.718504
chr10	88730351	88730745	AGAP11	179.782	178.944	17.5719	15.0995	132.852	127.185	20.1551	32.0616
chr10	88984256	88984494	NUTM2A	24.776	15.8873	0	0	17.4508	6.44659	0	0
chr10	89264955	89265248	MINPP1	138.193	155.339	6.6941	0.986266	223.258	172.28	17.142	34.1498
chr10	99080003	99080610	FRAT1	35.8357	24.9172	3.97693	1.19018	13.1959	12.6383	2.92041	0.49952
chr10	99474303	99474792	MARVELD1	366.584	348.82	20.672	16.5467	130.435	122.053	14.8026	7.13068
chr10	99475506	99475719	MARVELD1	108.891	96.6499	12.75	27.1339	61.9792	41.7787	20.1127	21.3527
chr11	117186452	117186759	BACE1	69.5713	78.7521	0.56379	2.15317	59.2374	41.6071	12.9334	3.47725
chr11	12696222	12696522	TEAD1	85.1745	100.831	7.04082	20.7099	155.254	148.826	26.5903	42.4492
chr11	128893826	128894026	ARHGAP32	151.401	89.7585	4.32709	11.5679	60.8832	38.4839	19.8528	24.9087
chr11	130784362	130784654	SNX19	502.294	471.004	42.0854	54.8967	301.654	261.345	40.2021	57.2754
chr11	1331509	1331709	TOLLIP-AS1	20.966	18.9059	0	0	8.89993	12.2743	1.47724	0
chr11	2719688	2719895	KCNQ1OT1	21.5231	29.0912	0.728863	0	15.0482	9.63563	6.42278	2.19717
chr11	288212	288437	PGGHG	46.5912	43.5691	0.670554	0.642169	13.8443	27.2762	0.656551	0.673797
chr11	43920692	43920892	SEC14L1P1	30.7939	18.9059	2.26312	2.88976	8.89993	22.2472	5.90896	11.3703
chr11	451568	451875	PTDSS2	33.7198	35.125	5.40594	4.70645	25.6078	28.9866	6.7366	5.92591
chr11	452147	452464	PTDSS2	83.5004	99.4001	2.85567	6.38117	47.2604	45.9803	4.66006	6.21722
chr11	45827563	45827760	SLC35C1	70.5076	49.7617	5.36103	4.40065	43.6714	42.8355	14.9974	18.4696
chr11	459065	459579	PTDSS2	15.5512	27.5183	1.46765	3.93547	8.9461	11.0445	2.58661	2.06465
chr11	536279	537718	LRRC56	89.6047	94.0113	6.08112	14.8605	44.2213	39.1302	10.1631	13.2746
chr11	62342276	62342477	EEF1G	20.2098	39.0171	0	0.718846	13.2835	6.86995	0	0
chr11	63606596	63606802	MARK2	48.9801	53.7061	0	2.80559	95.0478	137.788	27.2501	29.4378
chr11	65191335	65192171	NEAT1	2631.88	2573.55	344.702	353.788	1344.57	1325.07	398.996	346.55
chr11	65404257	65404935	MIR4690	568.023	544.064	15.577	13.2128	344.577	371.352	19.1736	12.0747
chr11	66113256	66113427	BRMS1	98.0251	48.2573	16.1949	8.69769	23.1196	95.7669	6.05728	34.3353
chr11	66114246	66114435	B4GAT1	91.5503	40.5974	3.66314	4.37185	67.7735	52.8542	21.9216	12.2378
chr11	73021089	73021380	LOC100287837	108.523	94.325	4.14776	3.47566	59.6387	60.1061	6.59935	4.16782
chr11	73021716	73022164	LOC100287837	54.4041	63.7699	4.04129	1.29007	71.8484	42.4669	11.5409	7.10646
chr11	75161911	75162104	GDPD5	14.0083	54.0082	0	1.7125	28.6779	72.9714	21.4673	5.53118
chr11	77524394	77524517	RSF1	45.0599	38.8412	4.22155	4.03064	69.4265	82.5464	43.509	27.4835
chr11	805364	805564	PIDD1	39.2021	58.6326	0.865418	0	14.2324	9.82569	10.358	0

chr11	85468758	85469076	SYTL2	204.47	223.076	13.0629	18.7082	36.3021	59.2219	2.71435	6.71394
chr12	109577406	109577746	ACACB	17.7286	14.0044	1.33125	0.424965	0.872542	0.902523	1.73793	1.33769
chr12	113772142	113772545	SLC8B1	94.2568	117.47	11.1667	6.9711	126.354	112.154	35.1262	9.27124
chr12	123736346	123736703	CDK2AP1	23.8552	25.1425	4.36345	2.31451	16.3897	9.17431	5.31921	7.97398
chr12	13155692	13155883	HTR7P1	18.5237	22.7296	1.57984	1.51296	2.32982	13.656	6.96082	2.38122
chr12	49319424	49319766	FKBP11	75.0998	75.3491	15.6889	9.18089	34.217	37.8279	19.6862	8.32372
chr12	51663014	51663480	SMAGP	143.012	117.433	25.9997	15.6036	87.8922	95.5861	38.8979	34.7439
chr12	56223939	56224662	DNAJC14	170.005	186.919	2.9215	5.19597	86.9887	79.1549	5.10802	10.9038
chr12	6716304	6716506	CHD4	87.666	67.3691	10.2822	1.6362	144.829	135.387	31.6208	9.68868
chr13	113663789	113664058	MCF2L	40.7047	87.7242	5.7909	10.4437	43.5027	32.2653	18.611	9.92116
chr13	41438822	41439022	MIR621	12.1662	19.5442	0.865418	0	4.74414	3.27523	1.72633	0
chr13	41766299	41766889	KBTBD7	60.2581	73.3674	2.93362	4.76161	60.0389	29.9767	5.85195	3.6187
chr13	42621599	42621874	DGKH	29.9853	8.94953	1.25879	0	7.47562	2.97748	0	0
chr13	45149279	45149910	TSC22D1-AS1	164.315	213.143	12.8921	19.9041	101.248	107.185	10.6698	14.9441
chr13	45910997	45911379	SNORA31	2907.07	2406.17	565.467	519.129	1728.76	1644.9	515.638	479.265
chr13	48986660	48986904	LPAR6	21.0526	31.4463	0	0	5.18486	11.4096	0.707511	0
chr13	52585706	52585928	ATP7B	36.5351	47.6052	0.779655	8.18836	46.3017	38.3585	3.88812	8.81583
chr13	77600836	77601139	FBXL3	45.9522	52.5574	1.14247	0.545399	101.772	165.923	11.9646	8.2207
chr13	95954179	95954432	ABCC4	42.4707	35.4261	0	1.7133	25.2106	12.7352	8.17445	0.599227
chr14	103291880	103292080	TRAF3	30.7939	81.2253	13.5787	13.0039	34.858	42.1929	29.5448	18.9506
chr14	103389594	103389969	AMN	41.4551	37.8337	5.07712	3.96614	9.27744	13.1009	4.14318	5.69342
chr14	105452939	105453276	CLBA1	64.5468	82.2809	15.6695	3.42998	26.4093	42.7962	12.7122	10.7968
chr14	105715669	105715920	BRF1	17.7726	27.6855	1.37915	3.29195	15.7508	25.445	10.3167	12.0503
chr14	24665697	24665950	TM9SF1	27.9685	55.9068	0	2.85549	9.38069	13.9481	2.33556	1.79768
chr14	34418491	34418704	EGLN3	241.166	212.06	101.575	69.8265	80.9251	72.2703	47.0079	42.6004
chr14	34418847	34419200	EGLN3	127.904	125.496	60.3096	32.3022	59.1338	42.2161	37.6564	17.6407
chr14	37641860	37642099	SLC25A21	57.0206	43.9468	0.631275	0	34.7556	15.4071	3.70855	0.634328
chr14	65878475	65878786	FUT8-AS1	38.6849	51.2055	2.22615	0.53137	8.64421	8.95159	1.66527	0
chr14	77491575	77492890	IRF2BPL	478.423	472.077	32.774	44.2358	387.829	390.91	38.4649	50.8728
chr14	93799026	93799295	BTBD7	39.6996	43.593	1.28687	1.22867	42.3269	50.5286	3.85054	7.27552
chr14	96831162	96831395	GSKIP	79.2975	42.0732	5.82778	3.1006	49.6562	40.1681	17.1182	17.5679
chr14	96968011	96968250	LOC730202	22.6242	24.2296	0	0	13.2333	11.6483	0	1.48887
chr15	101458391	101458826	LRRK1	11.498	14.3108	0	0	5.0895	3.38817	0	1.63604
chr15	101791768	101792063	CHSY1	42.616	45.6399	4.10707	2.80095	78.801	99.9222	12.2891	10.8561
chr15	22546439	22546660	REREP3	13.0445	15.2084	0	0	4.02712	2.08274	0	0
chr15	40212159	40212352	GPR176	30.1178	54.0082	0	0.856249	8.19368	15.2731	0	0
chr15	40861537	40861811	RPUSD2	66.9536	37.822	0.550638	1.05466	35.7296	50.9563	2.69569	6.08631
chr15	41099399	41100119	DNAJC17	90.0884	84.6097	0.838193	3.01017	41.2034	37.7179	5.1293	1.68449
chr15	43211934	43212244	TTBK2	64.9735	71.4519	9.49168	1.06617	23.9758	34.8653	4.45504	1.14787
chr15	45695390	45695807	SPATA5L1	76.6744	100.079	1.80905	2.07896	65.0954	65.1245	5.31381	5.81696
chr15	50647686	50648062	GABPB1	203.875	201.127	20.4644	19.5981	132.947	134.658	17.2868	19.3538
chr15	56207789	56208071	NEDD4	42.6631	50.3108	2.45509	2.34406	37.011	33.6814	4.89738	16.4039
chr15	63341096	63341296	TPM1-AS	40.5539	46.327	3.46167	0.82628	3.16276	3.27523	0	0

chr15	66995597	66996245	SMAD6	131.847	144.15	10.0117	4.45951	49.2151	52.5636	5.47126	1.87166
chr15	69488800	69489031	GLCE	34.0358	50.3187	0	5.00391	25.0431	22.5826	4.47648	6.56296
chr15	74753905	74754203	UBL7-AS1	48.8094	33.8361	4.05033	1.93944	12.444	15.4459	2.47859	4.57866
chr15	96875080	96875287	NR2F2	140.533	62.9182	27.6968	12.5642	37.9788	62.261	14.2728	18.3097
chr16	1016342	1016724	LMF1	43.8803	81.1026	3.17169	7.35433	33.5319	18.0052	11.7499	6.05485
chr16	1017388	1017656	LMF1	66.5811	70.2252	7.75001	8.63278	20.6524	29.9415	10.9506	7.96654
chr16	1822100	1822351	NME3	211.117	251.476	66.1993	90.1995	206.021	196.383	84.5969	111.288
chr16	2097587	2097787	NTHL1	25.0082	34.0214	2.59625	1.65256	45.0694	43.3968	8.63163	5.33758
chr16	21312364	21312656	CRYM-AS1	30.5156	20.1433	0	0.989644	20.8275	13.136	2.02362	3.63435
chr16	3063131	3063447	CLDN9	76.7151	120.987	1.43236	0	52.5734	26.2189	2.3374	0.479761
chr16	30905959	30906420	BCL7C	24.7295	17.9232	1.63639	0.313423	10.9399	7.6548	2.56354	0.32886
chr16	3535557	3535983	MIR6126	247.003	320.523	16.6458	23.403	150.77	179	16.645	17.0822
chr16	3702953	3703273	DNASE1	47.0917	56.8927	0	2.70915	56.5516	22.5349	1.38491	3.79011
chr16	4483175	4483384	DNAJA3	40.748	36.7125	5.79706	3.1628	8.32306	32.1254	4.95596	10.2155
chr16	57025952	57026327	NLRC5	29.003	33.9839	0.804665	3.46771	4.74663	20.4572	0	2.82995
chr16	58059790	58059970	MMP15	254.796	221.736	40.2333	48.9654	286.775	254.01	86.993	83.3824
chr16	67194159	67194433	FBXL8	62.1712	43.4442	7.15829	1.58199	38.9778	25.7581	6.46966	2.7665
chr16	67197738	67198151	HSF4	53.6208	73.9214	2.92251	2.44895	24.4227	30.4629	2.5038	1.8354
chr16	67978096	67978584	LCAT	325.484	363.71	28.3743	22.0116	222.949	301.683	27.5929	33.5422
chr16	69345355	69345555	VPS4A	74.6915	141.444	21.1225	15.1712	125.341	191.019	52.442	60.6418
chr16	70432470	70433328	ST3GAL2	215.216	263.559	11.902	12.3268	201.267	153.455	17.3035	9.74617
chr16	75298748	75298946	BCAR1	77.8307	95.0522	9.61575	34.2197	39.1352	31.429	13.9501	28.7547
chr16	770549	770757	FAM173A	11.0483	46.6332	0	0	9.12335	14.1717	9.12961	0
chr16	89786519	89787013	ZNF276	38.4625	43.0904	2.1379	4.97226	11.4102	19.8774	3.58844	5.21716
chr16	89986657	89987118	TUBB3	96.0754	136.702	3.60005	4.38792	61.4565	56.2462	4.16575	8.2215
chr16	90036306	90037004	AFG3L1P	104.387	116.357	3.96753	4.97189	47.5774	53.023	2.22592	3.31369
chr16	90038417	90038627	AFG3L1P	160.928	79.9692	4.94524	14.9517	86.5994	39.7706	3.28824	16.0975
chr17	14204308	14204536	HS3ST3B1	17.1939	17.779	0	0	8.32306	3.59126	0	0
chr17	18219159	18219425	SMCR8	118.722	81.0779	6.23918	2.71594	88.1071	72.6768	23.3248	11.9688
chr17	27401374	27401588	TIAF1	84.0136	95.3871	10.5144	10.8111	78.3301	100.246	20.1674	20.785
chr17	27894546	27895066	TP53I13	68.3697	29.2328	1.66426	0.3178	27.0659	31.1777	0.995957	1.02646
chr17	29151431	29151644	CRLF3	15.2315	43.4995	0	2.32755	16.3335	26.1403	6.48385	7.51772
chr17	30334849	30335052	LRRC37B	41.9579	68.987	2.9729	0	39.4578	61.9762	4.36623	2.98728
chr17	33289346	33289836	ZNF830	110.179	80.3108	5.85024	6.78209	73.2579	62.624	7.53694	8.97251
chr17	34958493	34958707	MRM1	65.5189	70.6763	4.93515	8.77731	15.2491	35.8479	19.3284	14.1686
chr17	3572702	3572980	EMC6	435.064	421.139	65.1258	50.9346	340.95	348.802	67.4854	77.4382
chr17	3629031	3629761	HASPIN	142.885	136.207	4.34023	4.1565	77.8236	90.1657	12.1417	5.81496
chr17	36859160	36859414	MIR4734	51.5897	34.7352	2.37598	0	15.7676	13.8932	2.90795	3.58121
chr17	3816836	3817071	P2RX1	17.8322	9.24076	0	0	18.1691	13.9371	1.46921	6.05683
chr17	40949748	40950011	COA3	225.128	155.781	9.87168	18.2221	138.897	107.099	13.7844	18.2655
chr17	41132867	41133406	RUNDC1	27.4717	32.7375	3.91882	1.6084	48.9854	50.6685	11.511	12.6571
chr17	43024582	43024936	KIF18B	55.7521	50.3021	2.44468	2.80095	33.0571	30.0692	15.1175	8.04156
chr17	43552637	43552877	MIR4315-1	132.363	146.582	15.866	17.2142	112.673	89.3864	17.9826	33.3599

chr17	46703577	46703917	HOXB9	140.348	173.3	19.3446	19.4419	143.255	134.381	46.7124	33.4907
chr17	57288304	57288962	SMG8	84.2385	84.7073	5.96162	7.46595	66.7269	70.4187	8.75568	12.2113
chr17	59489966	59490166	C17orf82	33.4146	33.6105	1.50875	0	6.67495	9.20573	0	0
chr17	62206446	62206973	ERN1	79.774	59.6119	3.28432	3.44937	25.8062	20.1983	6.55152	1.68804
chr17	656852	657052	DBIL5P	24.8972	16.8052	0	0	5.19162	6.13715	0.73862	0
chr17	72427482	72428097	GPRC5C	60.7248	58.0669	0.981299	1.1747	45.8262	45.405	1.92161	3.45116
chr17	7340444	7341233	FGF11	62.2803	81.2928	1.33856	6.59261	47.94	40.8366	3.93182	5.57228
chr17	76107255	76107538	TNRC6C-AS1	75.4714	56.2717	0.611602	0	56.438	39.9277	0.610009	3.14345
chr17	76107777	76108122	TNRC6C-AS1	38.7907	34.8292	0.501691	0.958006	24.7521	18.0375	3.50269	0.515709
chr17	79300928	79301156	TMEM105	36.7594	34.2881	0	0	26.3564	12.2103	134.775	0.780349
chr17	80376423	80376656	OGFOD3	39.3676	53.4931	5.18025	1.86036	49.0196	38.1926	13.9482	17.5679
chr18	11851772	11852274	CHMP1B	647.88	638.008	34.8635	28.2068	512.662	478.319	38.5495	30.8041
chr18	3449343	3449495	TGIF1	35.5736	40.9552	0	0	4.16153	8.61902	0	0
chr18	46476334	46477023	SMAD7	105.358	105.27	6.28024	11.0331	83.0856	85.0894	7.51666	19.6254
chr18	60190940	60191458	ZCCHC2	59.4476	57.3152	1.45632	0	42.0943	34.6548	1.71109	0.585345
chr18	60191600	60191754	ZCCHC2	61.2644	47.2875	4.89853	11.2588	34.675	13.9481	14.3887	7.87555
chr18	72347258	72347641	ZNF407	26.6865	23.7672	0.787858	0	30.2086	14.4215	2.69992	3.16667
chr18	72920845	72921024	ZADH2	46.0668	46.1005	2.90084	8.30896	19.436	43.9137	6.751	15.9034
chr18	9475566	9475833	RALBP1	114.351	87.0684	4.52059	5.95269	112.221	139.637	7.74583	17.602
chr19	10420823	10421182	ZGLP1	107.692	121.786	6.74977	14.27	115.85	75.2664	13.9453	17.8415
chr19	1206463	1207234	STK11	814.1	836.993	41.0943	51.7233	606.411	583.666	53.8397	54.4675
chr19	14183388	14183636	MISP3	22.8933	29.1879	0.697917	1.99907	21.6802	10.5653	5.56879	1.43483
chr19	16243940	16244200	HSH2D	290.803	325.332	49.3244	38.9006	170.011	204.769	52.8397	41.3997
chr19	17415424	17415685	MRPL34	83.342	69.217	5.78064	1.10719	30.1211	61.1364	6.22591	2.9043
chr19	17416112	17416387	MRPL34	105.307	154.812	2.74318	3.15247	67.4237	40.1705	4.8346	1.10258
chr19	17447615	17447972	GTPBP3	40.7428	44.3276	0.422618	2.02364	33.6552	28.365	2.89655	9.34257
chr19	19626103	19626353	TSSK6	54.6126	37.0616	3.46167	1.98307	22.1393	34.0624	1.38106	7.11678
chr19	2790227	2790438	THOP1	113.649	115.486	6.43542	6.163	93.4984	76.3509	9.10148	6.46654
chr19	33166825	33167106	RGS9BP	27.5133	20.4334	0.536921	0	2.63936	3.82207	0	0.539517
chr19	33167240	33167314	RGS9BP	67.2897	43.5271	0	0	6.01346	20.7336	1.99627	2.04871
chr19	33167857	33168067	RGS9BP	24.3356	26.675	0	2.06411	8.47612	3.65307	0	2.16578
chr19	33791953	33792544	CEBPA	408.97	568.799	19.622	32.436	244.03	230.541	17.5262	17.7618
chr19	33795108	33796039	CEBPA-DT	111.614	109.057	3.07908	4.3455	56.2419	50.5936	1.7454	4.23385
chr19	34663761	34663850	LSM14A	18.2265	39.0396	0	0	0	25.7602	3.87938	0
chr19	37006329	37006527	ZNF260	53.9353	59.2248	5.24495	1.66925	28.7524	77.7453	12.2063	17.9717
chr19	38045885	38046082	ZNF571-AS1	12.3514	6.61394	0	0.838863	27.2929	3.32511	7.88677	5.41887
chr19	39903519	39904137	PLEKHG2	803.825	742.821	33.4463	34.3685	432.035	392.013	27.7281	26.9846
chr19	4007761	4007975	PIAS4	67.3558	92.9262	2.11507	3.37589	29.8051	26.5274	7.59329	0.708432
chr19	47421915	47422557	ARHGAP35	1345.28	1174.01	62.277	52.2137	1005.98	923.68	65.5784	60.4529
chr19	47424947	47425139	ARHGAP35	589.67	531.728	65.2219	66.2237	368.513	462.684	70.7844	103.438
chr19	4792788	4793207	FEM1A	87.8797	91.9141	12.6029	8.27616	64.7847	71.771	13.0448	11.5784
chr19	47999026	47999262	NAPA-AS1	22.2098	21.956	0	0	7.54231	3.25061	2.5038	0
chr19	49206747	49207523	LOC105447645	194.193	175.957	14.7764	13.9647	105.132	110.524	10.4701	14.4571

chr19	50837533	50837812	KCNC3	43.2096	66.2572	0.54077	0.517878	30.8361	14.298	0.529477	0.543385
chr19	52308213	52308404	FPR3	32.5564	43.9621	0	0.865215	7.45153	15.433	0.903836	0
chr19	53236923	53237204	ZNF611	28.3829	28.8513	0	2.3524	34.8917	51.8675	14.1301	15.8291
chr19	56165540	56165804	U2AF2	49.6683	68.5473	0.655619	0.62597	54.5097	34.7373	3.26956	5.3915
chr19	57922331	57922522	ZNF17	40.4776	32.2614	3.9496	0	10.8725	24.9021	11.6014	3.9687
chr19	58363276	58363570	ZNF587	31.2661	45.3028	7.06463	5.05886	4.30308	7.79816	14.0925	7.26202
chr19	58952299	58952647	ZNF132	26.7347	34.6085	0	0.415195	9.80356	1.76355	0.424494	0
chr19	59031577	59031774	ZBTB45	45.2313	41.942	1.53172	1.46688	17.318	17.913	0.749868	0.769566
chr19	59082841	59083118	MZF1	182.517	158.883	13.1219	10.7387	105.616	108.189	20.5663	9.63463
chr19	8454827	8455182	RAB11B	75.0152	39.9652	2.4378	3.25857	35.1913	52.1269	4.3766	6.51536
chr20	18775374	18775583	LINC00652	5.64277	28.1428	0	0	5.67778	10.2775	1.41363	0
chr20	23345623	23346109	GZF1	102.457	63.9706	12.1072	6.24331	52.8014	64.4022	12.1584	17.1569
chr20	308531	308907	SOX12	858.715	895.386	47.7502	29.9736	197.25	173.016	16.5011	14.5153
chr20	3189621	3189821	ITPA	50.6924	79.6245	2.59625	0.82628	30.0462	15.5573	9.49479	15.1232
chr20	32320217	32320449	ZNF341	40.6669	37.4255	0.650322	1.24559	3.19681	1.98399	6.36741	0.653467
chr20	43559420	43559568	PABPC1L	213.379	237.507	60.146	57.6	267.599	316.188	103.806	114.728
chr20	44420646	44420810	DNTTIP1	35.9555	17.9324	0.919968	0	66.9303	50.5193	9.90832	2.77325
chr20	44518828	44519031	CTSA	42.6182	36.3713	3.41051	2.44221	10.9061	5.64695	1.70081	0
chr20	44992823	44993043	SLC35C2	165.902	201.364	0.786743	7.51164	69.7245	115.377	29.8184	17.7919
chr20	49575573	49575866	MOCS3	288.015	192.142	15.4479	20.2185	204.02	109.442	20.6713	22.2491
chr20	52198536	52198954	ZNF217	269.713	276.383	81.5728	58.1164	237.586	208.815	112.335	108.114
chr20	52199299	52199638	ZNF217	893.223	757.597	64.8425	40.4609	805.152	741.516	92.6818	82.9241
chr20	58514079	58514296	PPP1R3D	23.672	61.3779	0.79762	0	6.55872	5.28263	0	4.09953
chr20	61361346	61361850	NTSR1	45.0599	39.0654	0.34342	2.951	10.9818	16.2462	8.2206	8.82537
chr20	61368291	61368494	NTSR1	62.5955	25.6738	0	0.814069	24.1492	26.6213	1.70081	4.38225
chr20	61383238	61383540	LINC00659	98.9229	123.679	2.86562	10.3969	42.9382	59.1059	6.28794	6.48051
chr20	61885238	61885492	FLJ16779	9.28614	18.746	1.18799	0	0	0	0	0
chr20	61888395	61889312	NKAIN4	17.0049	23.8242	0.329062	0	0.485274	1.50584	0	0
chr20	6749106	6749536	BMP2	40.5303	28.0087	0	0.336019	27.2517	18.5542	0.343544	2.82055
chr20	746480	746700	SLC52A3	40.5539	42.7735	0	0.751164	33.7841	32.7523	6.27755	0.808725
chr21	33765880	33766240	URB1-AS1	35.6714	42.4021	7.96283	0.802711	4.53237	20.031	1.23103	2.10562
chr21	33800602	33800848	EVA1C	89.4892	50.6662	7.97306	3.5241	41.0024	23.7004	10.8091	13.5581
chr21	34922048	34922345	MIR6501	3255.65	3369.54	104.139	101.677	2614.54	2414.57	137.279	137.822
chr21	35467259	35467604	SLC5A3	89.6374	66.5715	9.18368	8.79492	46.0045	41.3591	17.1274	14.0619
chr21	37852605	37852749	CLDN14	21.8396	35.0109	0	0	3.09025	7.45835	0	0
chr21	37853171	37853373	CLDN14	23.3533	20.7986	0	0	5.14022	0.759549	0	0.750517
chr21	38633461	38633692	VPS26C	62.0305	67.0588	5.24495	5.72315	22.5912	38.9908	17.1885	7.70214
chr21	44867811	44868092	LINC00319	72.2806	69.7727	4.29537	8.22708	11.0853	11.4662	1.57712	2.69759
chr21	45936756	45936956	TSPEAR-AS2	32.4431	27.5067	0	0.82628	1.58138	9.82569	0	5.33758
chr21	46491280	46491480	SSR4P1	27.7118	20.2681	0	0	3.16276	0	0	0
chr21	46492611	46492777	SSR4P1	79.8049	46.2223	1.04267	0.995518	12.3843	4.93257	0	2.14361
chr21	46976077	46976277	SLC19A1	33.4146	45.5142	0	0.72244	6.67495	9.97288	0.73862	1.51604
chr21	47704040	47704430	MCM3AP	264.12	247.226	35.9481	38.5597	149.623	163.761	36.2971	45.6204

chr21	47742324	47742657	C21orf58	125.843	145.207	7.27678	0.992529	77.4069	63.9309	10.3683	0.534293
chr22	16155839	16156744	BMS1P22	112.027	90.8623	8.22386	12.2344	44.5583	36.7332	8.01168	7.47065
chr22	16157972	16158527	BMS1P22	46.5213	61.0389	11.5389	9.52828	19.6604	21.8349	8.39834	11.8613
chr22	21984456	21984625	YDJC	61.2543	53.863	6.24925	5.12975	25.4534	30.8673	6.11874	3.58827
chr22	31363779	31364271	MORC2	431.366	430.49	24.9775	33.5886	242.028	193.052	22.8072	30.3765
chr22	31366633	31366924	TUG1	418.78	347.944	25.405	12.9096	243.142	173.991	22.8439	21.881
chr22	31478260	31478535	SMTN	25.0697	21.0577	0	0	5.17543	5.95496	1.88326	2.58792
chr22	31743870	31744158	LINC01521	21.8396	22.3681	0	0.501695	4.12034	5.32739	0	2.10562
chr22	39873593	39874054	MGAT3-AS1	10.8014	22.4799	0	1.25369	2.57409	1.9969	1.28177	0.65772
chr22	39883430	39883634	MGAT3	35.9711	56.9786	0	0.708275	35.6288	9.02523	3.62069	0.743159
chr22	42475019	42475284	SMDT1	23.2407	40.1635	0	0.545238	5.59744	5.21079	1.1149	0.572092
chr22	43582728	43583003	TTLL12	94.38	101.077	4.40576	8.41303	135.711	163.166	26.3657	21.9973
chr22	46656037	46656320	PKDREJ	26.8558	32.6604	6.93064	1.02112	2.09657	10.3009	0	0
chrX	102003714	102004082	BHLHB9	48.427	33.4887	0.409986	0	16.5261	28.351	0.802848	0.411969
chrX	103216846	103217098	TMSB15B	17.6797	10.0031	0	0	3.53172	3.65307	1.75862	0
chrX	10534889	10535136	MID1	77.7146	69.1623	5.60594	5.35242	22.4082	27.8461	7.68809	5.04225
chrX	152617310	152617634	LOC105373378	64.3056	81.6921	1.39699	2.6757	35.2518	44.0398	1.36781	7.95455
chrX	48815489	48815855	OTUD5	35.0866	48.977	0	0	13.3742	14.253	0	4.1422
chrX	48932262	48932470	PRAF2	51.9922	72.3859	16.6426	5.5615	64.6238	81.8807	32.3686	22.2399
chrX	49644648	49645112	USP27X	83.593	108.353	3.25161	2.80257	54.9852	53.5678	6.04904	3.26734
chrX	49646011	49646389	USP27X	74.1854	129.3	1.59656	3.82243	41.9882	51.9547	3.90804	2.00535
chrX	54208915	54209141	FAM120C	40.0754	57.6525	5.36099	1.46244	22.3912	31.1581	4.58317	5.51078
chrX	56592009	56592240	UBQLN2	58.4281	42.4375	11.7565	5.00391	48.8019	50.4788	24.3009	13.7822
chrX	73070289	73070492	XIST	210.428	186.135	20.4631	43.1457	132.431	128.266	34.0163	42.0696
